# Highly Sensitive Flow Cytometry Allows Monitoring of Changes in Circulating Immune Cells in Blood After Tdap Booster Vaccination

**DOI:** 10.3389/fimmu.2021.666953

**Published:** 2021-06-10

**Authors:** Annieck M. Diks, Indu Khatri, Liesbeth E.M. Oosten, Bas de Mooij, Rick J. Groenland, Cristina Teodosio, Martin Perez-Andres, Alberto Orfao, Guy A. M. Berbers, Jaap Jan Zwaginga, Jacques J. M. van Dongen, Magdalena A. Berkowska

**Affiliations:** ^1^ Department of Immunology, Leiden University Medical Center, Leiden, Netherlands; ^2^ Leiden Computational Biology Center, Leiden University Medical Center, Leiden, Netherlands; ^3^ Cancer Research Centre (IBMCC, USAL-CSIC; CIBERONC CB16/12/00400), Institute for Biomedical Research of Salamanca (IBSAL), Salamanca, Spain; ^4^ Department of Medicine and Cytometry Service (NUCLEUS Research Support Platform), University of Salamanca (USAL), Salamanca, Spain; ^5^ Center for Infectious Disease Control, National Institute of Public Health and the Environment, Bilthoven, Netherlands; ^6^ Department of Hematology, Leiden University Medical Center, Leiden, Netherlands

**Keywords:** pertussis vaccine, flow cytometry, immune monitoring, plasma cells, correlation networks

## Abstract

Antigen-specific serum immunoglobulin (Ag-specific Ig) levels are broadly used as correlates of protection. However, in several disease and vaccination models these fail to predict immunity. In these models, in-depth knowledge of cellular processes associated with protective versus poor responses may bring added value. We applied high-throughput multicolor flow cytometry to track over-time changes in circulating immune cells in 10 individuals following pertussis booster vaccination (Tdap, Boostrix^®^, GlaxoSmithKline). Next, we applied correlation network analysis to extensively investigate how changes in individual cell populations correlate with each other and with Ag-specific Ig levels. We further determined the most informative cell subsets and analysis time points for future studies. Expansion and maturation of total IgG1 plasma cells, which peaked at day 7 post-vaccination, was the most prominent cellular change. Although these cells preceded the increase in Ag-specific serum Ig levels, they did not correlate with the increase of Ig levels. In contrast, strong correlation was observed between Ag-specific IgGs and maximum expansion of total IgG1 and IgA1 memory B cells at days 7 to 28. Changes in circulating T cells were limited, implying the need for a more sensitive approach. Early changes in innate immune cells, i.e. expansion of neutrophils, and expansion and maturation of monocytes up to day 5, most likely reflected their responses to local damage and adjuvant. Here we show that simultaneous monitoring of multiple circulating immune subsets in blood by flow cytometry is feasible. B cells seem to be the best candidates for vaccine monitoring.

## Introduction

Determination of antigen-specific immunoglobulin (Ag-specific Ig) levels in serum is routinely used as readout for vaccine efficacy and/or protective immunity (1–3). Besides Ag-specific Igs, immunological memory is preserved in the form of circulating memory B and T cells, which are more difficult to measure. These cells are preserved even when Ag-specific Ig levels have waned. Therefore, the cellular compartment may harbor potential for more accurate correlates of protection and provide insights into the mechanism of protection. Whereas serology provides insight in Ag-specific Ig levels and function, analysis of circulating immune cells may result in a deeper understanding of the processes induced by the vaccine and the cellular changes preceding Ig production. These additional insights can support the evaluation of novel vaccination strategies, such as addition of new adjuvants, antigens or changing the route of administration.

Cellular processes and their kinetics can be evaluated with different methods, such as ELISpot, cytokine production, tetramer staining or cell proliferation assays (4). These techniques have resulted in identification of several cellular correlates of protection. For example, Sridhar et al. reported that for flu a higher frequency of (pre-existing) cross-reactive IFNγ^+^IL-2^-^ CD8 T cells was associated with decreased disease symptoms, and CD45RA^+^CCR7^-^ late effector T cells within the above-mentioned cross-reactive T cells were a cellular correlate of protection (5). Furthermore, Wilkinson et al. showed increased numbers of influenza-specific CD4 T cells before the detectable increase in antibody levels (6). Despite their (generally) high sensitivity, such approaches may be laborious and require additional steps like pre-existing knowledge of HLA-type, prolonged incubation with or without culturing and stimulation, or isolation of cell subsets. Moreover, they mostly focus on a small part of the immune system and are therefore less suitable as an exploratory tool. Many of these limitations can be overcome with the use of flow cytometry or mass cytometry.

However, conventional flow and mass cytometry do have some limitations with regards to the monitoring of cellular processes and their kinetics in the blood. First, cells of interest can be present in low numbers in the peripheral blood (PB) [such as plasma cells, <5 cells/µL (7)], which may hamper their detection. This can be overcome by increasing sample volume, as applied in minimal residual disease monitoring (8). With the introduction of the new generation of high-speed flow cytometers, measuring increased cell numbers is becoming less of a hurdle. Second, cellular changes in PB may not directly reflect cellular changes in specific tissues. However, the blood stream is thought to be a ‘crossroad’ for cell trafficking. Leukocytes continuously circulate *via* blood through the body in search of damage or infection (9–11). This implies that when analyzed at the right time points, PB can contain valuable information about processes ongoing in the body (11–14). Flow cytometry can be an important tool in exploratory research, because it allows in-depth phenotyping and monitoring of millions of cells, while retaining information about absolute cell numbers. Finally, Ag-specific approaches are valuable tools, but not all antigens are commercially available, and associated costs can be high. Thus, it can be of interest to know which general changes can be observed post-vaccination.

A deeper understanding of cellular processes associated with vaccination may be of great value for pertussis research. The current acellular pertussis vaccine (aP) is a combined multivalent vaccine used to protect against tetanus, diphtheria and pertussis (Tdap) and, in some cases, additional diseases such as polio, Hib and hepatitis (15). It is mandatory or highly recommended in many countries, including the Netherlands (16, 17). Despite good vaccine coverage, the incidence of pertussis cases has increased over the past decennia (18). Therefore, an improved vaccination strategy or vaccine formulation based on in-depth understanding of cellular processes is of a great interest.

In this study, we used a pertussis booster vaccine (Tdap, Boostrix^®^, GlaxoSmithKline) as a model to extensively monitor cellular kinetics in the immune system of 10 healthy adults. Using high-dimensional flow cytometry, we investigated longitudinal changes in PB immune cell subsets before and after detectable increase in Ag-specific serum Igs. Moreover, we tested for correlations between total population kinetics and Ag-specific serum Ig levels. The exploratory nature of this study generated a vast amount of complex data, which is challenging to interpret without automated strategies. Therefore, we developed a top-down approach which starts with correlation network analysis to identify shared patterns between and within different immune cell populations. As the use of correlation network analysis yielded many correlations, we next evaluated the fluctuations of individual populations. Using this two-step approach, we assessed the complete dataset and identified most informative cell populations and time points post-Tdap booster vaccination. These can be further employed in larger scale studies, in order to e.g. evaluate candidate correlates of protection.

## Materials and Methods

### Study Design and Sample Collection

This study was approved by the Medisch-Ethische Toetsingscommissie Leiden-Den Haag-Delft (registration number: P16-214 EUDRACT: 2016-002011-18) and performed in competent adults after signing an informed consent form. Only volunteers who were (1) healthy, as evaluated by a questionnaire, (2) had blood hemoglobin levels and leukocyte differential counts within normal range, (3) had no suspected exposure to *Bp* in the past, (4) had a completed vaccination scheme according to Dutch National Immunization Program (www.rivm.nl/en/national-immunisation-programme) were eligible. Exclusion criteria are listed in **Supplementary Table 1**. Between June and December 2017, 10 individuals were included (m/f ratio: 1/9; age range: 25-55y, mean age: 37y), and completed the study. After initial blood collection (day 0), volunteers were vaccinated intramuscularly with the Boostrix^®^ vaccine (GlaxoSmithKline). This reduced-antigen, combined Tdap booster vaccine contains diphtheria toxoid (Diph) [2.5Lf (limit of flocculation)], tetanus toxoid (Tet) (5Lf), three *Bp* proteins -i.e. pertussis toxoid (PT) (8µg), filamentous hemagglutinin (FHA) (8µg), pertactin (Prn) (2.5µg) and aluminum hydroxide as adjuvant (19). PB samples were collected in K2EDTA blood collection tubes (BD Vacutainer, BD Biosciences, San Jose, CA, USA) and serum collection tubes (BD Vacutainer, BD Biosciences) at baseline (day 0) and subsequently at nine pre-defined time points i.e. day 3, 5, 7, 10, 14, 21 (20-21), 28 (28-31), 90 (90-95) and 1 year (day 363-371) post-vaccination. One donor was not eligible at the last time point.

### Evaluation of Total and Ag-Specific Serum Ig Levels

Serological analyses were performed in all collected samples. Levels of the three major Ig classes (IgM, IgA and IgG) were determined by turbidimetry, and IgG subclass (IgG1, IgG2, IgG3 and IgG4) levels were determined by nephelometry at the certified Clinical Chemistry laboratory at LUMC. Levels of IgG directed against Tet, Diph, PT, FHA, Prn and Fimbriae 2/3 (Fim2/3), and levels of IgA directed against PT, FHA, Prn and Fim2/3 were determined by multiplex immune assay (MIA) at the Dutch National Institute for Public Health and the Environment (RIVM, The Netherlands) (20).

### Longitudinal Flow Cytometric Analysis of up to 250 Circulating Immune Cells Subsets in Blood

All blood samples were subjected to high-throughput flow cytometric immunophenotyping with a panel of four recently developed multicolor immune monitoring antibody combinations (or their prototypes). In brief, the dendritic cell-monocyte panel (DC-Monocyte) allows analysis of up to 19 different (sub)populations within the myeloid compartment, including several subsets of monocytes and dendritic cells (21) (van der Pan et al., manuscript in preparation). The CD4 T-cell tube (CD4T) allows identification of at least 89 (up to 161) populations within the CD4 T-cell compartment with different functionalities and maturation stages, and longitudinal use of this tube may provide insight in the activation/maturation of T-cell subsets (21, 22). The CD8 cytotoxic T-cell tube (CYTOX) allows identification of up to 50 (sub)populations within the CD8 T-cell and the natural killer (NK) cell compartments (21). Lastly, the B-cell and plasma cell tube (BIGH) allows identification of up to 115 populations of B and plasma cells distinguished based on their maturation stage-associated phenotypic profile and the expressed Ig subclasses (7, 21).

Depending on the antibody combination, samples were either processed according to the bulk lysis protocol, for staining of 10 x 10^6^ cells (DC-Monocyte and BIGH) or prepared using the EuroFlow stain-lyse-wash protocol (CD4T, CYTOX; both protocols available on www.EuroFlow.org). For BIGH and CYTOX tubes, surface staining was followed by intracellular staining with the Fix & Perm reagent kit (Nordic MUbio, Susteren, The Netherlands) according to manufacturer’s protocol. In brief, 100µl of washed sample was fixed with 100µl of Solution A (15min in the dark at RT), washed, and permeabilized by adding 100µl of Solution B (15min in the dark at RT) and antibodies against intracellular markers. Next, samples were washed and re-suspended in PBS for immediate acquisition (or stored for max ~3h at 4°C). Additionally, BD TruCount tubes (BD Biosciences) were used according to manufacturer’s protocol for precise enumeration of cell subsets. By adding HLA-DR Pacific Blue, CD3 FITC, CD45 PerCP-Cy5.5, CD16 PE, CD56 PE, CD19 PE-Cy7, CD300e (IREM2) APC and CD14 APC-H7, we could determine absolute cell count of total leukocytes, eosinophils, neutrophils, monocytes (including classical, intermediate and non-classical monocytes [cMo, iMo and ncMo, respectively (23)], dendritic cells, basophils, total lymphocytes, B cells, NK cells and T cells. For each immune monitoring panel, a representative population was selected (e.g. total B-cell count as reference point in the BIGH panel) and used to determine absolute cell counts of all other populations in that panel. Immune monitoring tubes were measured on a BD FACS LSRFortessa 4L or BD FACS LSR Fortessa x20 4L flow cytometer (BD Biosciences, San Jose, CA, USA), while TruCount samples were measured on a BD FACS Canto™ II 3L (BD Biosciences) instrument. Flow cytometers were calibrated daily according to EuroFlow guidelines, as previously described (24, 25). All data were analyzed manually with Infinicyt software (Infinicyt™ Software v2.0, Cytognos) according to the gating strategies as proposed for these panels by EuroFlow (7, 22) (van der Pan et al., manuscript in preparation).

### Correlation Network Analysis

**Figure 1:**
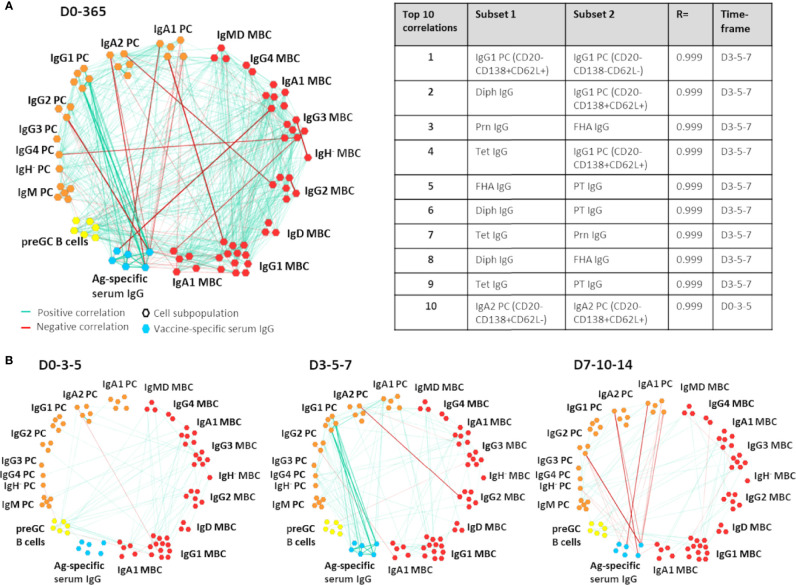
Example of Cytoscape network analysis between B-cell subsets and/or antigen-specific serum IgGs. In this example, correlations between and within the B-cell compartment and Ag-specific serum IgGs are shown. All positive and negative correlations with an R>0.90 or R<-0.90 over 3 sequential time points and present in at least 8/10 donors were visualized. Only subsets that had at least 1 correlation were shown in the network. For ease of interpretation, subset names have been removed and group names have been added. Of note, the most informative correlation networks found in this study were visualized in the Gephi software and are added as supplemental movies to this manuscript. MBC, Memory B cells (red); PC, plasma cells (orange); preGC B cells, pre-germinal center B cells (yellow); Ag-specific serum IgG, antigen-specific serum IgG (blue); D, Days after vaccination. **(A)** All correlations found within and between the B-cell compartment and Ag-specific serum IgGs at all time points. The table presents an overview of the 10 strongest correlations of the visualized network. **(B)** The most relevant correlations visualized per timeframe.

A sliding window of 3 time points was used to evaluate correlating changes between and within all immune subsets and Ag-specific Ig levels. To identify patterns shared by different individuals, the ratio over baseline was used as input. Pearson correlations were calculated using Hmisc library in R. The correlations were filtered based on their presence in at least 8/10 donors and the edges >90% positive and negative mean correlations were considered for visualization. To approach the data in a completely unbiased manner, we did not incorporate any adjustments or corrections for expected or implied correlations (e.g. when population A-B and B-C correlate strongly, a correlation between A-C is implied). For initial interpretation, the correlations were visualized in the open source software Cytoscape [version 3.7.1; Cytoscape Consortium (26)] (**Figure 1A**). To select the most informative time points, each timeframe was inspected individually (**Figure 1B**). The most relevant networks were selected and dynamic networks were generated using the open source Gephi software [version 0.9.2; Gephi.org (27)] and are attached to this manuscript as supplementary videos.

### Statistical Analysis of Independent Comparisons

Correlations of baseline levels of B cells, naive B cells, memory B cells (total, IgG1 and IgA1), plasma cells (total, IgG1, IgG4 and IgA1), total T cells, CD4 T cells, CD8 T cells, T follicular helper cells (TFHs), TCRγδ T cells, regulatory T cells (Tregs), naive CD4 T cells, NK cells, total leukocytes, neutrophils, monocytes, myeloid and plasmacytoid DCs (mDCs and pDCs) with the level (IU/mL) of vaccine-specific serum IgG at days 14, 21, 28, 90 and 365 were calculated using Spearman’s rank correlation.

For other statistical analyses (indicated in figure legends) the GraphPad Prism 8.1.1 software (GraphPad, San Diego, CA, USA), was used. Correlation coefficients (Spearman r) >0.8 were classified as strong correlations, and r-values <0.8 were classified as weak correlations. When multiple correlations were tested between two variables, but at several timepoints (e.g. maximum plasma cell expansion (day 7) and the level of vaccine-specific IgG (“Boostrix-IgG”) at days 14, 21 and 28), correction for multiple testing was done using the false discovery rate (FDR) approach method of Benjamini and Hochberg with an FDR of 5%.

## Results

### All Donors Reached Protective Serum Ig Levels 14 Days Post-Vaccination

Seven out of ten donors reported a painful/sore arm after receiving the vaccine. One serious adverse event, unrelated to this study, was reported.

Increase in Ag-specific serum Ig levels is the primary read-out of vaccine efficacy. To follow changes in serum Ig levels post-Boostrix^®^ vaccination and determine whether and when study participants reached protective levels, major Ig classes and IgG subclasses were measured at baseline and at all consecutive time points. In most donors, the levels of major Ig classes at baseline were within the normal reference ranges and followed subtle changes upon Boostrix^®^ vaccination (donor ranges: IgG 7.95-14.20 g/L, IgA 1.31-5.04 g/L, IgM 0.85-1.60 g/L, IgG1 4.35-8.87 g/L, IgG2 1.95-5.40 g/L, IgG3 0.30-0.53 g/L, IgG4 0.15-3.15 g/L) (**Supplementary Figure 1**) (28, 29).

**Figure 2:**
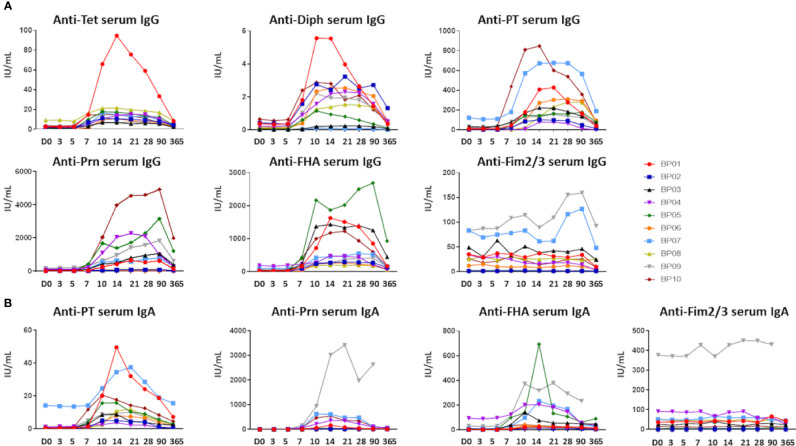
Ag-specific serum Ig levels prior to and post-aP booster vaccination. **(A)** Ag-specific serum IgG levels (IU/mL) directed against the 5 vaccine components (Diphtheria toxoid (Diph), tetanus toxoid (Tet), PT, FHA and Prn). Fim2/3 was not present in the vaccine and considered a negative control in **(A, B)**. **(B)** Ag-specific serum IgA levels (IU/mL) directed against the *Bp* vaccine components (PT, FHA, Prn). For Diph and Tet no quantitative read-out was available. D, Days after vaccination.

Ag-specific responses were analyzed separately for serum IgGs and IgAs (**Figures 2A, B** **)**. Baseline serum IgGs directed against Diph (median: 0.12 IU/mL, range: 0.007-0.65 IU/mL) were above protective levels [0.01-0.1 IU/mL (30–32)] in 5/10 donors, while baseline serum IgGs directed against Tet (median: 2.07 IU/mL, range: 0.29-9.30 IU/mL) were above protective levels (0.1 IU/mL (33)) in all donors. Baseline anti-PT serum IgGs (median: 11 IU/mL, range: 1-121 IU/mL) were at arbitrary protective levels of >20 IU/mL (34–36) in 3/10 donors. Baseline anti-FHA, anti-Prn and anti-Fim2/3 IgG levels were highly variable between donors (median anti-FHA: 33.5 IU/mL, range: 8-188 IU/mL, median anti-Prn: 20.5 IU/mL, range: 2-161 IU/mL, and lastly, median anti Fim2/3: 30 IU/mL, range: 1-83 IU/mL).

Baseline anti-PT and anti-Prn IgA levels (median anti-PT IgA: 0.56 IU/mL, range: 0.25-14.3 IU/mL, median anti-Prn IgA: 2.7 IU/mL, range: 0.67-12.7 IU/mL) were similar in most donors, whereas baseline anti-FHA and anti-Fim2/3 IgAs were detected at variable levels (median anti-FHA IgA: 4.0 IU/mL, range: 0.25-94.4 IU/mL, median anti-Fim2/3 IgA: 31.7 IU/mL, range 0.37-377.0 IU/mL). Elevated baseline levels of anti-PT IgG (121 IU/mL) and anti-PT IgA (14.3 IU/mL) in donor BP07 were either remaining high from previous vaccination or potential (subclinical) infection (37). In donor BP09, elevated anti-Prn and anti-Fim2/3, but no anti-PT IgAs were observed at baseline, indicative of previous contact with another microorganism, for example another *Bordetella* species (36).

In all donors, Ag-specific serum Ig levels started to rise from ~day 7 post-vaccination. Among *Bp* antigens the IgG level range at the peak was 79-847 IU/mL for anti-PT, 261-2688 IU/mL for anti-FHA and 39-4928 IU/mL for anti-Prn. For IgA the level range at the peak was 4-50 IU/mL for anti-PT, 14-693 IU/mL for anti-FHA and 6-3421 IU/mL for anti-Prn. As expected, for both Ig isotypes serum levels of anti-Fim2/3 Igs showed limited variation over time (max. ratio of 3x over baseline), since Fim2/3 was not a component of this vaccine. Although both Ig responses showed some heterogeneity in-between donors with regards to magnitude and number of targeted antigens, all donors reached arbitrary protective anti-PT IgG levels at day 14 post-vaccination the latest (>20 IU/mL). For diphtheria, all donors reached 0.01 IU/mL IgG levels (basic protection) the latest at day 7, and 0.1 IU/mL IgG levels (full protection) at day 10 (30–32). Waning was observed after 1 year for all vaccine-specific Ig responses. Nevertheless, 8/10 donors maintained protective anti-PT (>20 IU/mL) IgG and anti-Diph IgG (>0.1 IU/ml) serum levels at 1 year post-vaccination. All donors maintained protective anti-Tet IgG levels (>0.1 IU/ml) (33).

### Early Expansion of Plasma Cells Preceded the Increase in Vaccine-Specific Serum Ig Levels

All donors responded to vaccination by reaching protective IgG levels. To determine whether vaccination also triggered cellular changes which could be traced in circulation, we monitored the kinetics of over 250 immune cell populations in blood. Thereafter, we used correlation networks to investigate which cellular changes were associated with the change in Ag-specific Ig levels. Since not all identified correlations were biologically relevant (e.g. between two populations constant over time), we critically assessed these networks and used observed correlations as a guide to interpret the data.

**Figure 3:**
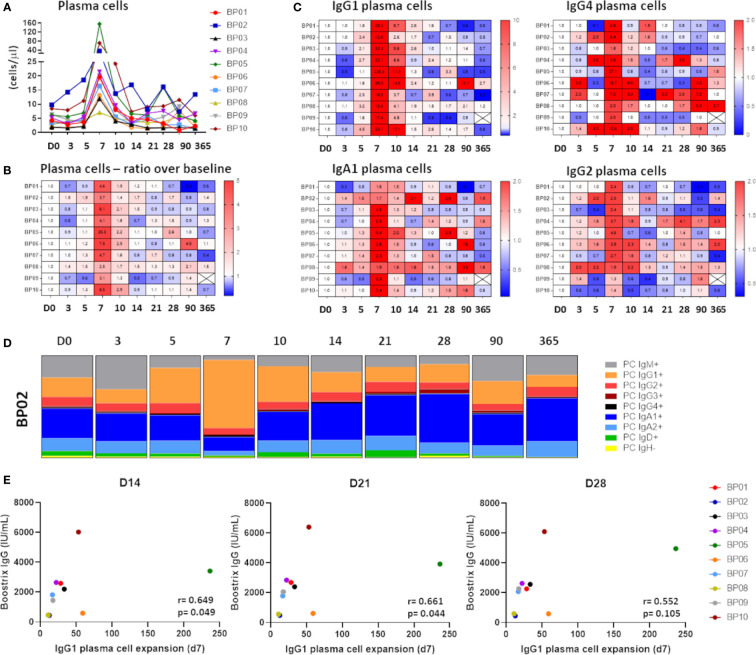
Kinetics in the plasma cell compartment upon immunization with aP booster. **(A)** Maximum expansion of plasma cells at day 7 (up to 156 cells/µl). Expansion started at day 5, peaked at day 7 and returned to baseline afterwards. **(B)** Heatmap representing the expansion of plasma cells in ratio over baseline. **(C)** Heatmaps representing the expansion of different plasma cell subsets. Only the most prominent expansions were visualized: IgG1 plasma cells (11-236x), IgG4 plasma cells (2-27x), IgA1 plasma cells (1.6-10.4x) and IgG2 plasma cells (1.3-4.6x). **(D)** Fluctuations in the distribution of the plasma cell compartment over time. One representative donor is shown. Majority of the expanded plasma cells was of IgG1 phenotype (min-max in donors: 44% -76%). **(E)** Correlation between maximum plasma cell expansion (day 7) and the level of vaccine-specific IgG (“Boostrix-IgG”) at days 14, 21 and 28 as determined by Spearman’s rank correlation. An FDR-corrected p-value of <0.0075 was considered significant. D, Days after vaccination.

Igs are the secretory product of terminally differentiated B cells (plasma cells). Therefore, we first focused on B-cell and plasma cell subsets and compared their kinetics with Ag-specific Igs within any 3-visit timeframes (**Dynamic network video: B-serology**). Plasma cells and Ag-specific serum Igs at multiple timeframes followed similar kinetics, as reflected by positive correlations between them. These correlations were the strongest between IgG1 and IgA1 plasma cells, and vaccine-specific Igs at day 3-5-7 (**Dynamic network video: B-serology**) due to the simultaneous rise of both Ig and plasma cell levels (**Figures 2**, **3A**). Interestingly, the more mature IgG1 plasma cells (CD20^-^CD138^-^) and IgA1 plasma cells (CD20^-^CD138^-^ and CD20^-^CD138^+^) showed stronger correlations with serum IgGs (anti-Tet, anti-Diph) and serum IgAs (anti-PT, anti-Prn and anti-FHA) (**Dynamic network video: B-Serology**) than their less mature counterparts. Furthermore, strong positive correlations were found between IgG4 plasma cells and Ag-specific serum IgGs (anti-Tet, anti-Diph, anti-Prn and anti-FHA) and IgAs (anti-FHA and anti-PT). At day 7-10-14, a negative correlation was observed between decreasing plasma cell numbers and increasing serum Ig levels (**Dynamic network video: B-Serology**). No correlations between plasma cells and Ag-specific Igs were observed at later time points i.e. day 21-365. Thus, correlations between kinetics of IgG1, IgA1 and IgG4 plasma cells and vaccine-specific serum Igs were not restricted to one particular vaccine antigen, but mainly limited to early timeframes post-vaccination.

Based on the change in the direction of correlation, we hypothesized that changes in plasma cell numbers precede changes in Ag-specific serum Igs. To test this hypothesis, we shifted the timeframes in Ag-specific Ig kinetics by 1, 2 or 3 time points later and earlier as compared to the plasma cells (**Supplementary Figure 2**). By shifting one timeframe later for serum Ig levels, strong positive correlations were found between IgG1 plasma cells (day 0-3-5) and vaccine-specific serum Ig levels (day 3-5-7), and between IgA1, IgG1 and IgG4 plasma cells (day 3-5-7) and vaccine-specific serum Ig levels (day 5-7-10), respectively (**Dynamic network video: B-Serology+1**). The number of edges and the correlation strength were similar as found when correlating within the same timeframes (**B-serology +1** *versus* **B-serology network**). Shifting the timeframe later by 2 visits resulted in fewer correlations. A strong positive correlation was observed between IgG1 plasma cells (day 0-3-5) and serum IgAs (anti-Prn and anti-PT; day 5-7-10), and between IgG4 plasma cells (day 7-10-14) and serum IgAs (anti-FHA; day 14-21-28), respectively (**Dynamic network video: B-Serology +2** **)**. Other timeframe comparisons i.e. 3 later and 1, 2 and 3 earlier timeframes did not reveal any relevant correlations. Thus, increasing plasma cell levels preceded for most of antigens the increase in serum Ig levels by 1-2 time points.

### Strong Increase in IgG1 Plasma Cell Levels did Not Explain Quantitative Changes in Ag-Specific Ig Levels

Strong correlations between plasma cell levels and Ag-specific Igs implied dynamic changes in the plasma cell compartment. Indeed, plasma cells showed a clear expansion from day 5 to day 14 post-vaccination, with a sharp peak at day 7 (ratio over baseline: 2.5-25x) (**Figures 3A, B** **)**. This was predominantly due to expansion of IgG1 plasma cells (ratio over baseline: 11-236x), followed by IgG4 plasma cells (ratio over baseline: 2-27x), IgA1 plasma cells (ratio over baseline: 1.6-9.4x), and IgG2 plasma cells (ratio over baseline: 1.3-4.6x) (**Figure 3C**). Expansion in other plasma cell subclasses was limited and restricted to individual donors (**Supplementary Figure 3**). Irrespective of the magnitude of changes, the distribution of plasma cells at day 7 was strongly skewed towards IgG1, which constituted 44%-76% of all plasma cells at the peak of expansion (**Figure 3D**).

Increase in plasma cell numbers in blood preceded and strongly correlated with an increase in Ag-specific serum Ig levels. Still, it remains unclear to what extent these circulating plasma cells are responsible for the entire vaccine-specific Ig production. To address this issue, we correlated the maximum IgG1 plasma cell expansion (day 7) with the vaccine-specific IgG levels measured at later time points (**Figure 3E**). Although donors with higher changes (ratio) in IgG1 plasma cells showed somewhat greater vaccine-specific IgG levels, no significant (strong) correlation was observed. Similarly, no statistical significance was reached between IgA1 or IgG4 plasma cell expansion (day 7) and vaccine-specific IgG levels (data not shown), or between any of the plasma cell subsets and vaccine-specific IgA levels.

### Circulating Plasma Cells Matured in Time Upon Immunization

**Figure 4:**
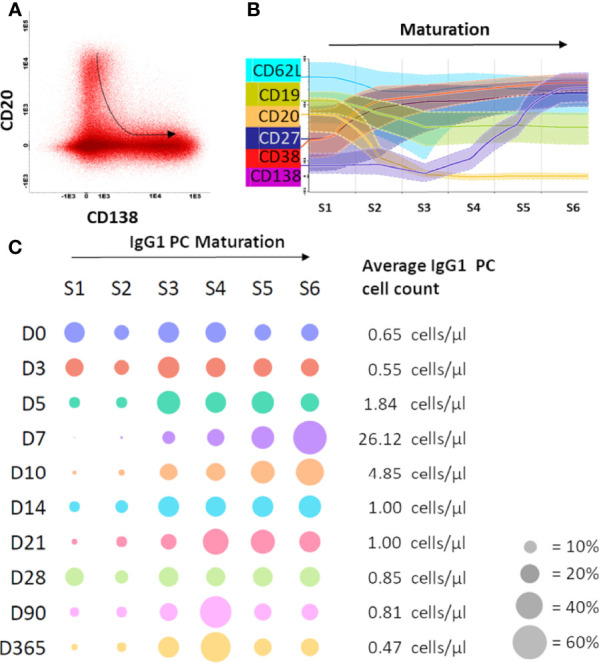
Maturation of IgG1 plasma cells was observed upon immunization with aP booster. **(A)** Flow cytometry files containing the plasma cells of all donors at all time points were merged in the Infinicyt software to visualize plasma cell maturation. The plasma cell maturation, defined by downregulation of CD20 and upregulation of CD138, is shown in the dot plot. The arrow indicates the direction of the maturation pathway. **(B)** Based on this CD20-CD138 maturation pathway, the Infinicyt software maturation tool identified 4 additional markers in the flow cytometry panel that were up- or downregulated upon plasma cell maturation. Based on the 6 identified markers (CD19, CD20, CD27, CD62L, CD38, CD138), 6 maturation stages were defined. **(C)** Per time point the percentage of plasma cells in each maturation stage was plotted (total IgG1 plasma cells of all donors, grouped per time point). The size of the dot indicates the percentage of plasma cells in a given maturation stage (average of 10 donors). Cell count is shown at the right side of the plot (average of 10 donors). The bubble plot was generated using plotly python graphing library. D, Days after vaccination.

Although plasma cell expansion preceded the increase in Ag-specific serum Ig levels, the magnitude of this expansion failed to explain quantitative changes in vaccine-specific serum Igs. This suggested that expanding plasma cells may be newly generated cells on their way to bone marrow or tissues (e.g. mucosal tissues). Newly generated plasma cells have high expression of CD20 and lack CD138. During maturation, CD20 is downregulated, which is followed by upregulation of CD138 at later stages (38) (**Figure 4A**). To investigate which other markers change their expression during plasma cell maturation, we drew a maturation pathway with the Infinicyt™ software maturation tool (21). Downregulation of CD20 was accompanied by milder downregulation of CD19 and CD62L, while upregulation of CD138 was preceded by increase in expression of CD38 and CD27 (**Figure 4B**). We used this information about the expression of all six markers to draw a new maturation pathway for IgG1 plasma cells and distinguished 6 consecutive maturation stages (S1-S6). Finally, the distribution of IgG1 plasma cells over the maturation stages was analyzed for samples collected at all 10 time points. At baseline and at day 3, IgG1 plasma cells were low in number, and relatively evenly distributed over all maturation stages (**Figure 4C**). From day 5 onwards, plasma cells expanded in numbers and more frequently belonged to more mature stages (S3-S6). At day 7, the peak of plasma cell expansion, the majority of IgG1 plasma cells reached the most mature phenotypes (~70% in S5+S6). In principle, the higher expansion of plasma cells, the more cells belonged to the most mature stages (**Supplementary Figure 4**). After day 7, plasma cell numbers and their distribution over maturation stages were gradually returning to baseline, which for individual donors was reached between days 14-28. The same phenomenon was observed in multiple other plasma cell subsets despite their overall lower expansion (IgG2, IgG3, IgG4, IgA1) (data not shown). Therefore, expanding plasma cells seem to be newly generated cells on their way to bone marrow, and this maturation observed in the periphery may be a hallmark of recent antigen encountering, such as vaccination. We further investigated this by correlating the absolute increase (cell count day 7 – baseline cell count) in IgG1 and IgA1 plasma cells (using the classical 3 maturation stages based on CD20 and CD138 expression) with the levels of Ag-specific Igs from day 7 onwards. In general, no strong correlations were found, with exception of the correlation between the absolute increase in CD20^-^CD138^-^ IgA1 plasma cells and the vaccine-specific IgG levels at day 7 (**Supplementary Figure 5**).

### Longitudinal Changes in Serum Ig Levels did Not Correlate With Longitudinal Changes in the Memory B-Cell Compartment

Despite clear expansion and maturation of circulating plasma cells, these failed to quantitatively explain expansion in Ag-specific Ig levels (**Figure 3E**), suggesting that most of the Ag-specific Igs are derived from memory responses (represented by long-lived plasma cells in bone marrow and memory B cells in periphery). Overall, fluctuations of memory B-cell numbers were limited over the time of analysis (ratio over baseline: 0.36-1.75x), as were fluctuations of total B cells and naive B cells (**Supplementary Figure 6**). In contrast to the plasma cell compartment, no skewing towards a particular memory B-cell subset was observed (data not shown).

Kinetics of IgG1 and IgA1 memory B-cell subsets strongly correlated with each other and, to a lesser extent, with IgG3 memory B-cell subsets. In contrast to plasma cells, correlations between Ag-specific Ig levels and memory B cells were absent both within and in-between timeframes (**Dynamic network videos: B-Serology**, **B-Serology +1**, **B-Serology +2**). Lastly, no correlation of baseline values of major B-cell populations and the vaccine-specific IgG levels at days 14, 21, 28, 90 or 365 was found (data not shown).

### Maximum Expansion of IgG1 and IgA1 Memory B Cells Correlated Strongly With the Increase in Ag-Specific Serum IgG

**Figure 5:**
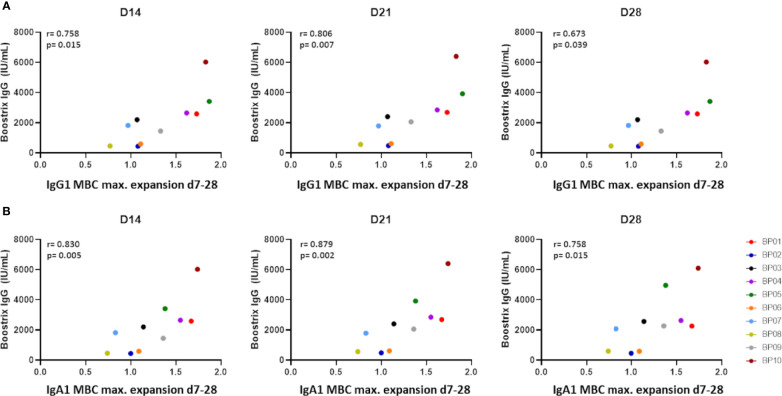
Correlation between maximum expansion of memory B-cell subsets and vaccine-specific IgGs as determined by Spearman’s rank correlation. **(A)** Correlation between the maximum expansion of IgG1 memory B cells (ratio over baseline at days 7-28) and vaccine-specific IgGs (“Boostrix-IgG”) at days 14, 21 and 28. **(B)** Correlation between the maximum expansion of IgA1 memory B cells (ratio over baseline at days 7-28) and vaccine-specific IgGs at days 14, 21 and 28. An FDR-corrected p-value of <0.0075 was considered significant. D, Days after vaccination.

Lack of correlations between Ag-specific serum Ig kinetics and memory B-cell kinetics is likely a consequence of the low frequency of expanding Ag-specific memory B cells in blood (39, 40). Furthermore, memory B-cell expansion may be less unified in time than plasma cell expansion. To address this second possibility, we correlated the levels of Ag-specific serum Igs at days 14-365 with the maximum observed expansion of total and individual memory B-cell subsets between days 7 and 28 (in majority of donors this maximum expansion was reached between days 7 and 14). In donors with a higher memory B-cell expansion there was a trend towards higher Ag-specific serum Ig, but no strong positive correlation (r>0.8) was found (data not shown). However, further investigation of memory B-cell subsets confirmed a strong positive correlation between IgG1 and IgA1 memory B-cell expansion (max. expansion days 7-28) and Ag-specific serum Ig. After correction for multiple testing, we found a strong positive correlation between total Ag-specific serum IgG levels at day 21 and maximum expansion of IgG1 memory B cells (r=0.8061, p=0.0072) (**Figure 5A**). Furthermore, the maximum expansion of IgA1 memory B cells strongly correlated with serum IgG levels at days 14 and 21 (r=0.8303, p=0.0047 and r=0.8788, p=0.0016, respectively) (**Figure 5B**). These strong correlations between IgG1 and IgA1 memory B-cell expansions and the increased Ag-specific serum IgG levels could not simply be explained by changes in total B-cell or leukocyte numbers (**Supplementary Figure 7** **)** and were stronger than any correlation between serum IgG levels and the day 7 IgG1 plasma cell peak (**Figure 3E**). As the rise in Ag*-*specific Igs was mostly caused by *Bp*-specific Igs, the abovementioned correlations held true when correlating only to *Bp*-specific Ig levels, but not for Diph-specific or Tet-specific serology (data not shown).

### Minor Changes in Circulating T Cells Showed Limited Correlations With B Cells and Ag-Specific Igs

T-cell help in the germinal centers results in the generation of high affinity B cells (41). For Tdap vaccination, polarization of CD4 T-cell responses towards Th2 has been described (42). However, recent studies indicated that in donors who were wP-primed, T-cell responses upon aP booster vaccinations were skewed towards a Th1/Th17 response (43, 44). Within CD4 T cells, we evaluated both kinetics of different T-helper cell types (including TFHs and Tregs) and of different effector stages within these subsets (e.g. naive, central-, transitional-, peripheral- and effector memory).

Analysis of correlation networks revealed multiple correlations in-between CD4 T-cell subsets within the same timeframes. These correlations were especially prominent between T helper and TFH cells of the TH17, TH1/17 and CXCR3^+^CCR4^+^CCR6^+^CCR10^-^ phenotype. At the same time, no consistent strong correlations were found between CD4 T cells and Ag-specific Igs, and correlations between CD4 T cells and B cells were limited and restricted to minor populations (**Dynamic network video: B-CD4**). Despite several correlations, changes in absolute T-cell subset numbers over time were limited (**Supplementary Table 2**). Likewise, no consistent changes were observed in maturation of T helper subsets. Thus, in this case, monitoring of T-cell kinetics in the periphery may require an Ag-specific approach.

Additionally, we correlated CD8 T-cell and NK-cell kinetics with changes in B cells and Ag-specific Ig levels. Within the CD8 T-cell and NK-cell compartments correlations were limited and inconsistent, suggesting no shared response pattern. Likewise, hardly any correlations were observed between both Ag-specific Igs and NK or CD8 T cells, and between B cells and NK or CD8 T cells (mostly restricted to CD8 or TCRγδ T-cell subsets). When comparing CD4 T-cell kinetics with NK and CD8 T-cell kinetics, very limited or no correlations were observed. Lastly, no correlation of baseline values of major cell populations and the vaccine-specific IgG levels at days 14, 21, 28, 90 or 365 was found (data not shown).

### Changes in Innate Immune Cells Preceded, but Poorly Correlated With Changes in the T- or B-Cell Compartments

So far, post-vaccination kinetics of serum Igs, B cells and T cells were evaluated over time, but not the kinetics of innate immune cells. Local damage, antigens and adjuvant introduced by the vaccine lead to the recruitment of innate immune cells, which serve an important role in initiating the immune response by means of local inflammation and antigen presentation (45–48).

Overall, correlations between kinetics of innate immune cells and the change in Ag-specific serum Ig levels were limited and mostly restricted to monocyte subsets [cMo, iMo and ncMo, further subdivided into different functional subsets/activation stages, e.g. based on expression of CD62L, FcER1, CD36 or SLAN (49, 50) (van den Bossche & Damasceno et al., manuscript in preparation)]. Likewise, correlations between kinetics in the innate immune cell compartment and the B-compartment were sparse, both within and in-between timeframes. No correlations were found between T-cell and innate immune cell fluctuations within the same timeframes (**Dynamic network video: CD4- DC Monocyte**), and only limited correlations were present upon shifting timeframes (early monocyte changes with later Treg changes). Lastly, no correlations between baseline values of major innate immune cell populations and the vaccine-specific IgG levels at days 14, 21, 28, 90 or 365 were found (data not shown).

In terms of absolute cell counts, in approximately half of the donors, an increase in total leukocyte count was observed at the earliest evaluated time points post-vaccination (days 3-5, **Supplementary Figure 8A**). This was mainly due to an increase in both mature and immature neutrophils (total neutrophils: max. ratio over baseline: 2.6x) (**Supplementary Figure 8B**). At the same time – mostly in donors that showed neutrophil expansion - total monocyte numbers showed a max. ratio over baseline of 2x and at day 3 predominantly belonged to the more mature iMos and ncMos. No increase was observed for cMos.

## Discussion

**Figure 6:**
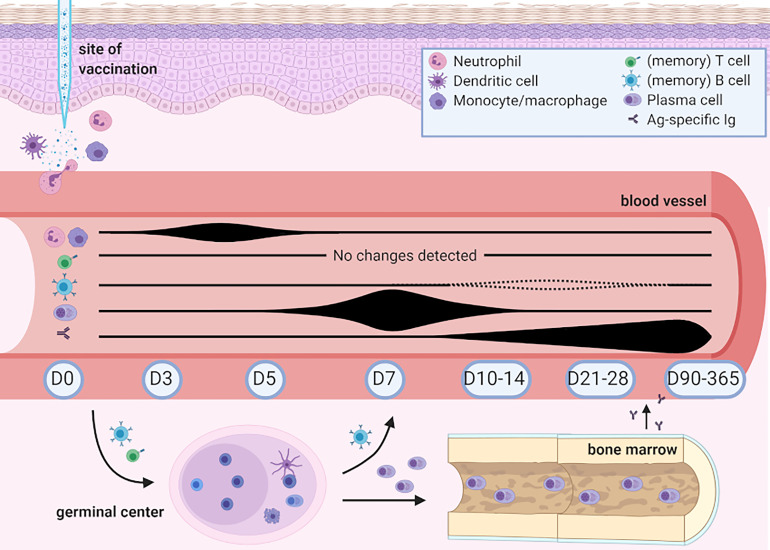
Overview of changes detected in human peripheral blood post-Boostrix vaccination in this study. Created with BioRender.com. Up to 5 days post-vaccination, fluctuations were found in the levels of circulating neutrophils and monocytes, with a change in total monocyte composition (based on expression of CD14 and CD16, with increased levels of intermediate and non-classical monocytes). Circulating plasma cells started expanding from day 5 onwards with a sharp peak in (predominantly IgG1) plasma cell levels at day 7 simultaneous with plasma cell maturation. Changes in circulating memory B-cell numbers were limited. Increase in Ag-specific IgG serum levels occurred from day 7 onwards and only showed signs of waning at day 365. Despite in-depth analysis, no uniform changes were detected in circulating T-cell subsets. Baseline cell count and Ag-specific serum Ig levels did not seem to influence the levels of Ag-specific IgGs.

In this study we showed that flow cytometry is a sensitive tool to monitor cellular changes upon vaccination. Although most of these changes occur locally in the tissue, many cells can be traced during their passage in PB, if sufficiently sensitive methods are applied. Up to 5 days post-Tdap-vaccination, fluctuations were predominantly found in the levels of neutrophils and monocytes and were associated with gradual maturation of monocytes. Afterwards, plasma cells started expanding from day 5 onwards with a sharp peak in (predominantly IgG1) plasma cell levels at day 7, simultaneously with plasma cell maturation. Despite limited changes in memory B-cell numbers, these changes strongly correlated with increase in Ag-specific serum IgG levels, which occurred from day 7 onwards. Although memory B cells seem better correlated with serological responses, strong homogenous plasma cell increase and clear plasma cell maturation over time can play a valuable role in immune response timing. Despite in-depth analysis, no uniform changes were detected in circulating T-cell subsets. We summarize our findings and their potential role in immune monitoring in **Figure 6**.

Correlation networks are frequently used in systems biology to model kinetic relationships between different types of omics data (51–53). Analysis of correlation networks is suited for large, multidimensional datasets, and can aid in finding shared or correlating patterns in exploratory studies. Such results should be analyzed cautiously as two interacting pairs that remain constant over time will correlate as well, which may be irrelevant for the posed research question. Therefore, identified correlations should be confirmed by experimental data or in another group of samples. In our study, we correlated longitudinal changes in circulating immune cells and serology readouts over a 3 time point window.

Increase in number of neutrophils, iMo and ncMo was the earliest signs of immune response to Boostrix. In fact, innate immune cell kinetics might be even more prominent than observed as we did not study cellular changes before day 3. Mouse models have shown increased numbers of neutrophils and monocytes at the site of vaccination already within hours (54). Moreover, upon Ebola vaccination, a significant increase in frequency and activation state of DCs and monocyte subsets was observed after 1 and 3 days, and a gene enrichment set analysis after flu vaccination pointed towards gene signatures from innate immunity modules after 1 day (55, 56). The changes observed in our study may not be vaccine specific, but rather related to the local damage or adjuvant (11). It has been shown in man and mouse that the type of adjuvant used and the route of administration can influence the innate response (54, 57). Lastly, Rechtien et al. showed a correlation between innate markers and post-vaccination antibody levels (days 28 and later) (55). Therefore, flow cytometric analysis of innate immune cells at early time points (days 1-3) may be a valuable tool to evaluate different adjuvants as well as different routes of vaccine administration.

Increase in plasma cell numbers started at day 5 and peaked at day 7 post-vaccination. In steady state, numbers of circulating plasma cells in blood are low, with median values in adults of <5 cells/µL (7), in contrast, plasma cell counts expand rapidly upon infection or vaccination. This highly dynamic behavior brings both challenges and opportunities. Because of low baseline numbers, reliable detection of circulating plasma cells has only become possible upon introduction of high-throughput flow cytometry. In turn, the system is relatively ‘clean’ and easy to study without the need of introducing an antigen-specific approach. It has been shown in an influenza model that at the peak of expansion up to 80% of the generated IgG plasma cell peak can be antigen specific (12, 58). The same is likely to be true in our Tdap booster study. This is in contrast to memory B cells, where cells raised in response to the vaccine or infection may constitute <0.1% of all B cells (59, 60). Currently, we are evaluating which of the components of the multivalent Boostrix vaccine are recognized by most Ag-specific B cells, and whether there are phenotypical differences in-between plasma cells with different specificities.

At the peak of expansion majority of plasma cells was of the IgG1 phenotype. Ag-specific serum Igs were mostly IgGs and correlated well with the max. expansion of IgG1 memory B cells (max. ratio over baseline between days 7-28). In fact, IgGs (mainly IgG1) are the most frequently raised in response to vaccination and for many vaccines Ag-specific serum IgGs are considered correlates of protection (61, 62). IgG1 plays an important role in complement activation and antibody dependent cell-mediated cytotoxicity (63). Moreover, IgG1 can be transferred to the placenta to protect fetus and newborns, which can be highly relevant for maternal vaccination programs (64, 65).

Increase in Ag-specific serum IgA levels and IgA B cells was less pronounced. This can be related to the fact that -in contrast to IgG- IgA responses are mostly associated with natural infections and occur locally in the mucosa-associated lymphoid tissue. Moreover, the monomeric serum IgAs -generated upon vaccination- are metabolized at a faster rate than serum IgGs (66). Therefore, serum IgA levels are rarely considered a read-out of vaccine efficacy, i.e. in case of rotavirus (67). The volunteers in this study were adults. As *Bp* carriership within the population is high, it is likely that they have encountered *Bp* earlier in life and had pre-existing IgA memory B cells which became activated upon antigen encounter (68, 69). Indeed, several studies showed an age-dependent increase in *Bp*-specific serum IgA levels (70, 71). Alternative vaccine administration routes, such as the intranasal delivery of a life-attenuated bacterium in the BPZE1 vaccine, can also result in robust Ag-specific IgA responses (57).

The composition of the plasma cell compartment in steady state is a mixture of plasma cells of different maturation stages (72), and over the course of an immune response, the contribution of the most mature plasma cells (CD20^-^CD138^+^) increases. Since the magnitude of plasma cell expansion differs between donors, knowledge of these maturation stages may be useful when assessing the progress of an immune response. Although up to 80% of total plasma cells at the peak of expansion can be Ag-specific and derived from the ongoing response, the origin of remaining plasma cells may be somewhat different. Odendahl and colleagues showed that upon vaccination with tetanus toxoid a considerable amount of circulating plasma cells, not specific to the used vaccine, were long-lived plasma cells forced to leave their niche in the bone marrow upon competition with newly generated plasma cells (13). These cells may further contribute to the observed shift in plasma cell maturation. Moreover, the competition for the bone marrow niche prolongs retention of the newly generated plasma cells in the blood, which may start maturing before leaving circulation.

The early expansion of plasma cells at days 5-7 was closely followed by an increase in Ag-specific serum Igs from day 7 onwards. This rise in serum Igs continued even when plasma cell numbers had returned to baseline. However, magnitude of plasma cell expansion did not reflect quantitative changes in serum Ig levels. One of the potential explanations of this phenomenon is that, at the peak of response, part of the circulating plasma cells may be derived from the “expelled” long-lived plasma cells in bone marrow rather than from the ongoing immune response (13). Further explanation may have to do with plasma cell affinity. If affinity of the newly produced Abs is low, they may not be detected in Ag-specific assays, such as MIA. Finally, it remains unclear whether all produced plasma cells will be able to successfully home to bone marrow and start Ig production. To shed more light on these issues, it would be of value to compare the B-cell receptors (BCR) of circulating plasma cells with the structure of Ag-specific Igs.

Despite minor quantitative changes, and in contrast to circulating plasma cells, total IgG1 memory B cells at the peak of expansion (max. ratio over baseline between days 7-28) correlated well with Ag-specific serum Ig levels (IU/mL). Correlations between Ag-specific memory B cells and serum Ig levels, have been observed for tetanus toxoid and rotavirus, but were not corroborated by other studies on tetanus toxoid and wasp venom (59, 60). The different techniques used in-between those studies may account for the different findings. A study on pertussis by Hendrikx et al. showed correlations between circulating memory B cells as measured by ELISpot and *Bp*-specific serum Igs (at baseline with FHA, post-vaccination with FHA, PT and Prn) (73). Although these techniques yield valuable data, they are laborious and difficult to apply in daily practice. Our non-Ag-specific approach, when confirmed in a larger cohort, would be more convenient in a way that it is not dependent on the availability and costs of (labeled) antigens.

Although the CD4 T-cell panel used in this study allowed us to monitor different maturation and activation stages of (minor) T-cell subsets in a highly sensitive manner, no consistent kinetics or maturation of T cells were detected in this study. However, a recent study by Lamberts et al., showed that their recently developed T-cell assay enabled relatively fast detection of Ag-specific T-cell kinetics upon aP vaccination (44). Similarly, Da Silva and colleagues were able to monitor an Ag-specific CD4 T-cell response upon aP vaccination (43). Still, both studies showed low prevalence of Ag-specific T cells, which could explain the limited cellular changes observed in the T-cell compartment in our approach.

Of note, the magnitude of cellular changes differed between donors. This may be due to individual differences in the immune system responsiveness and previous (natural) *Bp* exposure, as also visible from diverse Ig levels generated post-vaccination. Furthermore, it can be related to differences in timing of the response. In our study, we included days 5, 7 and 10 and observed a clear plasma cell peak at day 7. This is in line with previous studies using rabies, tetanus and influenza vaccination, which reported detection of plasma cells 6-7 days after secondary immunization (12, 14, 74). However, somewhat delayed responses in some of the donors cannot be excluded. Moreover, timing may differ in case of a primary immunization or when using a different route of delivery, therefore the findings presented in this study may not directly be extrapolated to all types of vaccination (14, 57).

Lastly, 2/10 donors in this study did not have protective anti-PT serum IgG levels (>20 IU/mL) 1 year after vaccination. These two donors did not show a deviating cellular response as compared to the other donors. Moreover, a decrease in Ag-specific Ig levels 1 year after vaccination was observed for all donors. Whether these donors are still protected, may not only depend on the Ag-specific antibodies, but also on the presence of memory cells (75).

In this study, we implemented a broad in-depth flow-cytometric approach to determine the most relevant time points for cellular immune analysis in vaccination studies and to identify candidate populations for novel cellular correlates of protection. This approach could be useful in early evaluation of e.g. vaccine candidates, altered routes of vaccine/antigen administration or the setup of disease models. Currently, we are comparing cellular kinetics post-Boostrix vaccination in this cohort and additionally in four cohorts of different ages and priming backgrounds, with cellular kinetics post-bacterial challenge in humans, as recently described by De Graaf and colleagues (76, 77). This work is done within the framework of the IMI (Innovative Medicines Initiative) PERISCOPE Consortium (https://periscope-project.eu/). In these studies, additional analyses are performed by different IMI PERISCOPE partners providing insights into Ag-specific immunity, local (mucosal) immunity, cytokine and chemokine production (44, 78, 79). This should yield novel insights into the extent to which current vaccines mimic naturally obtained immunity.

## Data Availability Statement

The raw data supporting the conclusions of this article will be made available by the authors, without undue reservation.

## Ethics Statement

The studies involving human participants were reviewed and approved by Medisch-Ethische Toetsingscommissie Leiden-Den Haag-Delft. The patients/participants provided their written informed consent to participate in this study.

## Author Contributions

MB, JD, and LO designed the study. LO and JJZ coordinated the clinical part of this study. GB coordinated the Ag-specific serology work at RIVM. BM, RG, AD, CT, and MB performed the experimental work and data analysis. AO and MP-A provided conceptual input. IK performed bioinformatics analysis and constructed the correlation networks. AD and MB wrote the manuscript. All authors contributed to the article and approved the submitted version.

## Funding

IK is supported by the European Union’s Horizon 2020 research and innovation program under the Marie Skłodowska-Curie grant agreement No 707404. The here presented study is a pilot study for the Innovative Medicines Initiative (IMI) PERISCOPE program, a Joint Undertaking under grant agreement No 115910. This Joint Undertaking receives support from the European Union’s Horizon 2020 Research and Innovation Programme, the European Federation of Pharmaceutical Industries and Associations (EFPIA), and the Bill and Melinda Gates Foundation (BMGF). The flow cytometric studies in this study were supported by the EuroFlow Consortium. The EuroFlow Consortium received support from the FP6-2004-LIFESCIHEALTH-5 program of the European Commission (grant LSHB-CT-2006-018708) as Specific Targeted Research Project (STREP).

## Conflict of Interest

AD, CT, JD, AO, MP-A and MB report inventorship of the patent “Means and methods for multiparameter cytometry-based leukocyte subsetting” (NL2844751, filing date 5 November 2019) (21), owned by the EuroFlow Consortium.

The remaining authors declare that the research was conducted in the absence of any commercial or financial relationships that could be construed as a potential conflict of interest.

## References

[1] Plotkin SA. Complex Correlates of Protection After Vaccination. J Clin Infect Dis (2013) 56(10):1458–65. 10.1093/cid/cit048 23386629

[2] Whittle H Inskip H Hall A Mendy M Downes R Hoare S. Vaccination Aginst Chronic Viral Carriage in The Gambia. Lancet (1991) 337(8744):747–50. 10.1016/0140-6736(91)91367-4 1672389

[3] Plotkin SA. Correlates of Protection Induced by Vaccination. Clin Vaccine Immunol (2010) 17(7):1055–65. 10.1128/CVI.00131-10 PMC2897268 20463105

[4] Clay TM Hobeika AC Mosca PJ Lyerly HK Morse MA. Assays for Monitoring Cellular Immune Responses to Active Immunotherapy of Cancer. J Clin Cancer Res (2001) 7(5):1127–35. 11350875

[5] Sridhar S Begom S Bermingham A Hoschler K Adamson W Carman W. Cellular Immune Correlates of Protection Against Symptomatic Pandemic Influenza. Nat Med (2013) 19(10):1305. 10.1038/nm.3350 24056771

[6] Wilkinson TM Li CK Chui CS Huang AK Perkins M Liebner JC. Preexisting Influenza-Specific CD4+ T Cells Correlate With Disease Protection Against Influenza Challenge in Humans. Nat Med (2012) 18(2):274. 10.1038/nm.2612 22286307

[7] Blanco E Pérez-Andrés M Arriba-Méndez S Contreras-Sanfeliciano T Criado I Pelak O. Age-Associated Distribution of Normal B-Cell and Plasma Cell Subsets in Peripheral Blood. J Allergy Clin Immunol (2018) 141(6):2208–19.e16. 10.1016/j.jaci.2018.02.017 29505809

[8] Theunissen P Mejstrikova E Sedek L van der Sluijs-Gelling AJ Gaipa G Bartels M. Standardized Flow Cytometry for Highly Sensitive MRD Measurements in B-Cell Acute Lymphoblastic Leukemia. Blood J Am Soc Hematol (2017) 129(3):347–57. 10.1182/blood-2016-07-726307 PMC5291958 27903527

[9] Springer TA. Traffic Signals for Lymphocyte Recirculation and Leukocyte Emigration: The Multistep Paradigm. Cell (1994) 76(2):301–14. 10.1016/0092-8674(94)90337-9 7507411

[10] Butcher EC Picker LJ. Lymphocyte Homing and Homeostasis. Science (1996) 272(5258):60–7. 10.1126/science.272.5258.60 8600538

[11] van den Bossche WB Rykov K Teodosio C Ten Have BL Knobben BA Sietsma MS. Flow Cytometric Assessment of Leukocyte Kinetics for the Monitoring of Tissue Damage. Clin Immunol (2018) 197:224–30. 10.1016/j.clim.2018.09.014 30290225

[12] Ellebedy AH Jackson KJ Kissick HT Nakaya HI Davis CW Roskin KM. Defining Antigen-Specific Plasmablast and Memory B Cell Subsets in Human Blood After Viral Infection or Vaccination. Nat Immunol (2016) 17(10):1226. 10.1038/ni.3533 27525369 PMC5054979

[13] Odendahl M Mei H Hoyer BF Jacobi AM Hansen A Muehlinghaus G. Generation of Migratory Antigen-Specific Plasma Blasts and Mobilization of Resident Plasma Cells in a Secondary Immune Response. Blood (2005) 105(4):1614–21. 10.1182/blood-2004-07-2507 15507523

[14] Blanchard-Rohner G Pulickal AS Jol-van der Zijde CM Snape MD Pollard AJ. Appearance of Peripheral Blood Plasma Cells and Memory B Cells in a Primary and Secondary Immune Response in Humans. Blood (2009) 114(24):4998–5002. 10.1182/blood-2009-03-211052 19843885 PMC2788974

[15] CDC. Vaccines & Preventable Diseases Home. [CDC Website] (2020). Available at: https://www.cdc.gov/vaccines/vpd/dtap-tdap-td/hcp/about-vaccine.html.

[16] Schurink-van’t Klooster T De Melker H. The National Immunisation Programme in the Netherlands: Surveillance and Developments in 2015-2016. RIVM Open Reposit (2016) 151–62. 10.21945/RIVM-2019-0193

[17] Girard DZ. Recommended or Mandatory Pertussis Vaccination Policy in Developed Countries: Does the Choice Matter? Public Health (2012) 126(2):117–22. 10.1016/j.puhe.2011.11.007 22226337

[18] Tan T Dalby T Forsyth K Halperin SA Heininger U Hozbor D. Pertussis Across the Globe: Recent Epidemiologic Trends From 2000 to 2013. Pediatr Infect Dis J (2015) 34(9):e222–32. 10.1097/INF.0000000000000795 26376316

[19] GSK. Boostrix- Highlights of Prescribing Information: Glaxosmithkline. Available at: https://www.gsksource.com/pharma/content/dam/GlaxoSmithKline/US/en/Prescribing_Information/Boostrix/pdf/BOOSTRIX.PDF.

[20] van Gageldonk PG van Schaijk FG van der Klis FR Berbers GA. Development and Validation of a Multiplex Immunoassay for the Simultaneous Determination of Serum Antibodies to Bordetella Pertussis, Diphtheria and Tetanus. J Immunol Methods (2008) 335(1-2):79–89. 10.1016/j.jim.2008.02.018 18407287

[21] van Dongen JJM Orfao de Matos Correia E Vale JA Goncalves Grunho Teodosio CI Perez Y Andres M. Means and Methods for Multiparameter Cytometry-Based Leukocyte Subsetting. Octrooicentrum Nederland, Den Haag. P119646NL00 (2019).

[22] Botafogo V Pérez-Andres M Jara-Acevedo M Bárcena P Grigore G Hernández-Delgado A. Age Distribution of Multiple Functionally Relevant Subsets of CD4+ T Cells in Human Blood Using a Standardized and Validated 14-Color EuroFlow Immune Monitoring Tube. Front Immunol (2020) 11:166. 10.3389/fimmu.2020.00166 32174910 PMC7056740

[23] Ziegler-Heitbrock L Ancuta P Crowe S Dalod M Grau V Hart DN. Nomenclature of Monocytes and Dendritic Cells in Blood. Blood (2010) 116(16):e74–80. 10.1182/blood-2010-02-258558 20628149

[24] Kalina T Flores-Montero J Lecrevisse Q Pedreira CE Velden VH Novakova M. Quality Assessment Program for EuroFlow Protocols: Summary Results of Four-Year (2010–2013) Quality Assurance Rounds. Cytometry Part A (2015) 87(2):145–56. 10.1002/cyto.a.22581 25345353

[25] Kalina T Flores-Montero J Van Der Velden V Martin-Ayuso M Böttcher S Ritgen M. EuroFlow Standardization of Flow Cytometer Instrument Settings and Immunophenotyping Protocols. Leukemia (2012) 26(9):1986–2010. 10.1038/leu.2012.122 22948490 PMC3437409

[26] Shannon P Markiel A Ozier O Baliga NS Wang JT Ramage D. Cytoscape: A Software Environment for Integrated Models of Biomolecular Interaction Networks. Genome Res (2003) 13(11):2498–504. 10.1101/gr.1239303 PMC403769 14597658

[27] Bastian M Heymann S Jacomy M, eds. Gephi: An Open Source Software for Exploring and Manipulating Networks. In: Third International AAAI Conference on Weblogs and Social Media. Proceedings of the International AAAI Conference on Web and Social Media (2009). Available at: https://ojs.aaai.org/index.php/ICWSM/article/view/13937.

[28] Schauer U Stemberg F Rieger CH Borte M Schubert S Riedel F. Igg Subclass Concentrations in Certified Reference Material 470 and Reference Values for Children and Adults Determined With the Binding Site Reagents. Clin Chem (2003) 49(11):1924–9. 10.1373/clinchem.2003.022350 14578325

[29] The German Society for Clinical Chemistry and the Association of Diagnostics-Industry. E.V. V. Konsensuswerte Der Deutschen Gesellschaft Für Laboratoriumsmedizin. Clin Lab (1995) 41:743–8.

[30] Ipsen J. Circulating Antitoxin at the Onset of Diphtheria in 425 Patients. J Immunol (1946) 54(4):325–47. 20278364

[31] Edmunds W Pebody R Aggerback H Baron S Berbers G Conyn-van Spaendonck M. The Sero-Epidemiology of Diphtheria in Western Europe. Epidemiol Infect (2000) 125(1):113–25. 10.1017/S0950268899004161 PMC2869577 11057967

[32] Galazka A. Diphtheria: The Immunological Basis for Immunisation. Geneva: World Health Organization (1993). WHO/EPI/GEN/93.12.

[33] Edsall G. Specific Prophylaxis of Tetanus. J Am Med Assoc (1959) 171(4):417–27. 10.1001/jama.1959.73010220003012 13819371

[34] Hendrikx LH Felderhof MK Öztürk K de Rond LG van Houten MA Sanders EA. Enhanced Memory B-cell Immune Responses After a Second Acellular Pertussis Booster Vaccination in Children 9 Years of Age. Vaccine (2011) 30(1):51–8. 10.1016/j.vaccine.2011.10.048 22064265

[35] Long SS Welkon CJ Clark JL. Widespread Silent Transmission of Pertussis in Families: Antibody Correlates of Infection and Symptomatology. J Infect Dis (1990) 161(3):480–6. 10.1093/infdis/161.3.480 2313126

[36] Guiso N Berbers G Fry NK He Q Riffelmann M von König CW. What to do and What Not to do in Serological Diagnosis of Pertussis: Recommendations From EU Reference Laboratories. Eur J Clin Microbiol Infect Dis (2011) 30(3):307–12. 10.1007/s10096-010-1104-y PMC3034915 21069406

[37] de Melker H Versteegh F Conyn-van Spaendonck M Elvers L Berbers G van Der Zee A. Specificity and Sensitivity of High Levels of Immunoglobulin G Antibodies Against Pertussis Toxin in a Single Serum Sample for Diagnosis of Infection With Bordetella Pertussis. J Clin Microbiol (2000) 38(2):800–6. 10.1128/JCM.38.2.800-806.2000 PMC86208 10655388

[38] Perez-Andres M Paiva B Nieto WG Caraux A Schmitz A Almeida J. Human Peripheral Blood B-cell Compartments: A Crossroad in B-cell Traffic. Cytometry Part B: Clin Cytometry (2010) 78(S1):S47–60. 10.1002/cyto.b.20547 20839338

[39] Nanan R Heinrich D Frosch M Kreth HW. Acute and Long-Term Effects of Booster Immunisation on Frequencies of Antigen-Specific Memory B-Lymphocytes. Vaccine (2001) 20(3-4):498–504. 10.1016/S0264-410X(01)00328-0 11672915

[40] Buisman A De Rond C Öztürk K Ten Hulscher H Van Binnendijk R. Long-Term Presence of Memory B-Cells Specific for Different Vaccine Components. Vaccine (2009) 28(1):179–86. 10.1016/j.vaccine.2009.09.102 19799844

[41] Gatto D Brink R. The Germinal Center Reaction. J Allergy Clin Immunol (2010) 126(5):898–907. 10.1016/j.jaci.2010.09.007 21050940

[42] Bancroft T Dillon MB da Silva Antunes R Paul S Peters B Crotty S. Th1 Versus Th2 T Cell Polarization by Whole-Cell and Acellular Childhood Pertussis Vaccines Persists Upon Re-Immunization in Adolescence and Adulthood. Cell Immunol (2016) 304:35–43. 10.1016/j.cellimm.2016.05.002 27212461 PMC4899275

[43] da Silva Antunes R Babor M Carpenter C Khalil N Cortese M Mentzer AJ. Th1/Th17 Polarization Persists Following Whole-Cell Pertussis Vaccination Despite Repeated Acellular Boosters. J Clin Invest (2018) 128(9):3853–65. 10.1172/JCI121309 PMC6118631 29920186

[44] Lambert EE Corbière V van Gaans-van den Brink J Duijst M Venkatasubramanian PB Simonetti E. Uncovering Distinct Primary Vaccination-Dependent Profiles in Human Bordetella Pertussis Specific CD4+ T-Cell Responses Using a Novel Whole Blood Assay. Vaccines (2020) 8(2):225. 10.3390/vaccines8020225 PMC7349943 32429152

[45] Li H Nookala S Re F. Aluminum Hydroxide Adjuvants Activate Caspase-1 and Induce IL-1β and IL-18 Release. J Immunol (2007) 178(8):5271–6. 10.4049/jimmunol.178.8.5271 17404311

[46] Coffman RL Sher A Seder RA. Vaccine Adjuvants: Putting Innate Immunity to Work. Immunity (2010) 33(4):492–503. 10.1016/j.immuni.2010.10.002 21029960 PMC3420356

[47] Lu F HogenEsch H. Kinetics of the Inflammatory Response Following Intramuscular Injection of Aluminum Adjuvant. Vaccine (2013) 31(37):3979–86. 10.1016/j.vaccine.2013.05.107 23770306

[48] Lindblad EB. Aluminium Compounds for Use in Vaccines. Immunol Cell Biol (2004) 82(5):497–505. 10.1111/j.0818-9641.2004.01286.x 15479435

[49] Hamers AA Dinh HQ Thomas GD Marcovecchio P Blatchley A Nakao CS. Human Monocyte Heterogeneity as Revealed by High-Dimensional Mass Cytometry. Arterioscler Thromb Vasc Biol (2019) 39(1):25–36. 10.1161/ATVBAHA.118.311022 30580568 PMC6697379

[50] Damasceno D Almeida J Teodosio C Sanoja-Flores L Mayado A Pérez-Pons A. Monocyte Subsets and Serum Inflammatory and Bone-Associated Markers in Monoclonal Gammopathy of Undetermined Significance and Multiple Myeloma. Cancers (2021) 13(6):1454. 10.3390/cancers13061454 33810169 PMC8004952

[51] Adourian A Jennings E Balasubramanian R Hines WM Damian D Plasterer TN. Correlation Network Analysis for Data Integration and Biomarker Selection. Mol Biosyst (2008) 4(3):249–59. 10.1039/b708489g 18437268

[52] Hood L Perlmutter RM. The Impact of Systems Approaches on Biological Problems in Drug Discovery. Nat Biotechnol (2004) 22(10):1215–7. 10.1038/nbt1004-1215 15470453

[53] Kelder T Stroeve J Bijlsma S Radonjic M Roeselers G. Correlation Network Analysis Reveals Relationships Between Diet-Induced Changes in Human Gut Microbiota and Metabolic Health. Nutr Diabetes (2014) 4(6):e122–e. 10.1038/nutd.2014.18 PMC4079927 24979151

[54] Liang F Loré K. Local Innate Immune Responses in the Vaccine Adjuvant-Injected Muscle. Clin Trans Immunol (2016) 5(4):e74. 10.1038/cti.2016.19 PMC4855268 27195117

[55] Rechtien A Richert L Lorenzo H Martrus G Hejblum B Dahlke C. Systems Vaccinology Identifies an Early Innate Immune Signature as a Correlate of Antibody Responses to the Ebola Vaccine Rvsv-ZEBOV. Cell Rep (2017) 20(9):2251–61. 10.1016/j.celrep.2017.08.023 PMC5583508 28854372

[56] Nakaya HI Clutterbuck E Kazmin D Wang L Cortese M Bosinger SE. Systems Biology of Immunity to MF59-adjuvanted Versus Nonadjuvanted Trivalent Seasonal Influenza Vaccines in Early Childhood. Proc Natl Acad Sci (2016) 113(7):1853–8. 10.1073/pnas.1519690113 PMC4763735 26755593

[57] Lin A Apostolovic D Jahnmatz M Liang F Ols S Tecleab T. Live Attenuated Pertussis Vaccine BPZE1 Induces a Broad Antibody Response in Humans. J Clin Invest (2020) 130(5)2332–46. 10.1172/JCI135020 PMC7190984 31945015

[58] Wrammert J Smith K Miller J Langley WA Kokko K Larsen C. Rapid Cloning of High-Affinity Human Monoclonal Antibodies Against Influenza Virus. Nature (2008) 453(7195):667. 10.1038/nature06890 18449194 PMC2515609

[59] Rojas OL Narváez CF Greenberg HB Angel J Franco MA. Characterization of Rotavirus Specific B Cells and Their Relation With Serological Memory. Virology (2008) 380(2):234–42. 10.1016/j.virol.2008.08.004 PMC2582161 18789807

[60] Leyendeckers H Odendahl M Löhndorf A Irsch J Spangfort M Miltenyi S. Correlation Analysis Between Frequencies of Circulating Antigen-Specific IgG-bearing Memory B Cells and Serum Titers of Antigen-Specific Igg. Eur J Immunol (1999) 29(4):1406–17. 10.1002/(SICI)1521-4141(199904)29:04<1406::AID-IMMU1406>3.0.CO;2-P 10229109

[61] Schure R-M Hendrikx LH de Rond LG Öztürk K Sanders EA Berbers GA. Differential T-and B-Cell Responses to Pertussis in Acellular Vaccine-Primed Versus Whole-Cell Vaccine-Primed Children 2 Years After Preschool Acellular Booster Vaccination. Clin Vaccine Immunol (2013) 20(9):1388–95. 10.1128/CVI.00270-13 PMC3889587 23825195

[62] Robbins JB Schneerson R Szu SC. Perspective: Hypothesis: Serum IgG Antibody is Sufficient to Confer Protection Against Infectious Diseases by Inactivating the Inoculum. J Infect Dis (1995) 171(6):1387–98. 10.1093/infdis/171.6.1387 7769272

[63] Parham P. The Immune System; Ch.9 Immunity Mediated by B Cells and Antibodies. 711 Third Avenue, New York, USA and Abingdon, UK: Garland Science, Taylor & Francis Group, LLC (2014).

[64] Safadi MAP. Control of Pertussis in Infants: Time has Finally Come? Expert Rev Vaccines (2015) 14(6):781–3. 10.1586/14760584.2015.1043274 25968349

[65] RIVM. Pertussis- 22-Week Vaccination [Website of the RIVM]. Available at: www.rivm.nl.

[66] Woof JM Kerr MA. The Function of Immunoglobulin A in Immunity. J Pathol (2006) 208(2):270–82. 10.1002/path.1877 16362985

[67] Franco MA Angel J Greenberg HB. Immunity and Correlates of Protection for Rotavirus Vaccines. Vaccine (2006) 24(15):2718–31. 10.1016/j.vaccine.2005.12.048 16446014

[68] Cherry JD. Epidemic Pertussis in 2012—the Resurgence of a Vaccine-Preventable Disease. New Engl J Med (2012) 367(9):785–7. 10.1056/NEJMp1209051 22894554

[69] van der Maas HdM N Heuvelman K van Gent M Mooi FR. Kinhoestsurveillance in 2013 En 2014 - RIVM Briefrapport 2014-0165. Rijksinstituut voor Volksgezondheid en Milieu (RIVM), kinkhoestsurveillance in 2013 en 2014 (2014). Available at: https://rivm.openrepository.com/handle/10029/557163.

[70] Subissi L Rodeghiero C Martini H Litzroth A Huygen K Leroux-Roels G. Assessment of IgA Anti-PT and IgG Anti-ACT Reflex Testing to Improve Bordetella Pertussis Serodiagnosis in Recently Vaccinated Subjects. Clin Microbiol Infect (2020) 26(5):645.e1–8. 10.1016/j.cmi.2019.10.001 31610300

[71] Hendrikx LH Öztürk K De Rond LG De Greeff SC Sanders EA Berbers GA. Serum IgA Responses Against Pertussis Proteins in Infected and Dutch Wp or Ap Vaccinated Children: An Additional Role in Pertussis Diagnostics. PloS One (2011) 6(11):e27681. 10.1371/journal.pone.0027681 22110718 PMC3215732

[72] Caraux A Klein B Paiva B Bret C Schmitz A Fuhler GM. Circulating Human B and Plasma Cells. Age-associated Changes in Counts and Detailed Characterization of Circulating Normal CD138– and CD138+ Plasma Cells. Haematologica (2010) 95(6):1016–20. 10.3324/haematol.2009.018689 PMC2878802 20081059

[73] Hendrikx LH de Rond LG Öztürk K Veenhoven RH Sanders EA Berbers GA. Impact of Infant and Preschool Pertussis Vaccinations on Memory B-Cell Responses in Children at 4 Years of Age. Vaccine (2011) 29(34):5725–30. 10.1016/j.vaccine.2011.05.094 21669247

[74] Frölich D Giesecke C Mei HE Reiter K Daridon C Lipsky PE. Secondary Immunization Generates Clonally Related Antigen-Specific Plasma Cells and Memory B Cells. J Immunol (2010) 185(5):3103–10. 10.4049/jimmunol.1000911 20693426

[75] Hendrikx LH Öztürk K De Rond LG Veenhoven RH Sanders EA Berbers GA. Identifying Long-Term Memory B-Cells in Vaccinated Children Despite Waning Antibody Levels Specific for Bordetella Pertussis Proteins. Vaccine (2011) 29(7):1431–7. 10.1016/j.vaccine.2010.12.033 21187178

[76] De Graaf H Gbesemete D Gorringe AR Diavatopoulos DA Kester KE Faust SN. Investigating Bordetella Pertussis Colonisation and Immunity: Protocol for an Inpatient Controlled Human Infection Model. BMJ Open (2017) 7(10):e018594. 10.1136/bmjopen-2017-018594 PMC5652574 29025851

[77] de Graaf H Ibrahim M Hill AR Gbesemete D Vaughan AT Gorringe A. Controlled Human Infection With Bordetella Pertussis Induces Asymptomatic, Immunising Colonisation. Clin Infect Dis (2019) 71:403–11. 10.1093/cid/ciz840 PMC7353841 31562530

[78] Versteegen P Pinto MV Barkoff AM van Gageldonk PG van de Kassteele J van Houten MA. Responses to an Acellular Pertussis Booster Vaccination in Children, Adolescents, and Young and Older Adults: A Collaborative Study in Finland, the Netherlands, and the United Kingdom. EBioMedicine (2021) 65:103247. 10.1016/j.ebiom.2021.103247 33647770 PMC7920834

[79] iavatopoulos DA Mills KH Kester KE Kampmann B Silerova M Heininger U. PERISCOPE: Road Towards Effective Control of Pertussis. Lancet Infect Dis (2019) 19(5):e179–86. 10.1016/S1473-3099(18)30646-7 30503084

