# Supplementary material for: Highly Sensitive Flow Cytometry Allows Monitoring of Changes in Circulating Immune Cells in Blood After Tdap Booster Vaccination
Source: Front Immunol. 2021 Jun 10;12:666953. doi: 10.3389/fimmu.2021.666953 (PMC8223751; doi:10.3389/fimmu.2021.666953)
Supplement: Supplementary file 1 [file DataSheet_1.docx]

Supplementary Material

**Supplementary Tables can be found in the downloaded ‘Supplementary Materials’**

**Supplementary Table 1:** Exclusion criteria for this study.

**Supplementary Table 2:** Interactive table in MS Excel which allows visualization of the fluctuations of all major cell populations assessed in this study. Excel file Tab 1: B-cell compartment, Tab 2: T-cell compartment and NK cells, and Tab 3: Innate immune cell compartment.

**Supplementary videos can be found in the downloaded ‘Supplementary Materials’:**

Video 1: Dynamic network video: B- CD4

Video 2: Dynamic network video: B-Serology

Video 3: Dynamic network video: B-Serology +1

Video 4: Dynamic network video: B-Serology +2

Video 5: Dynamic network video: CD4-DC Monocyte


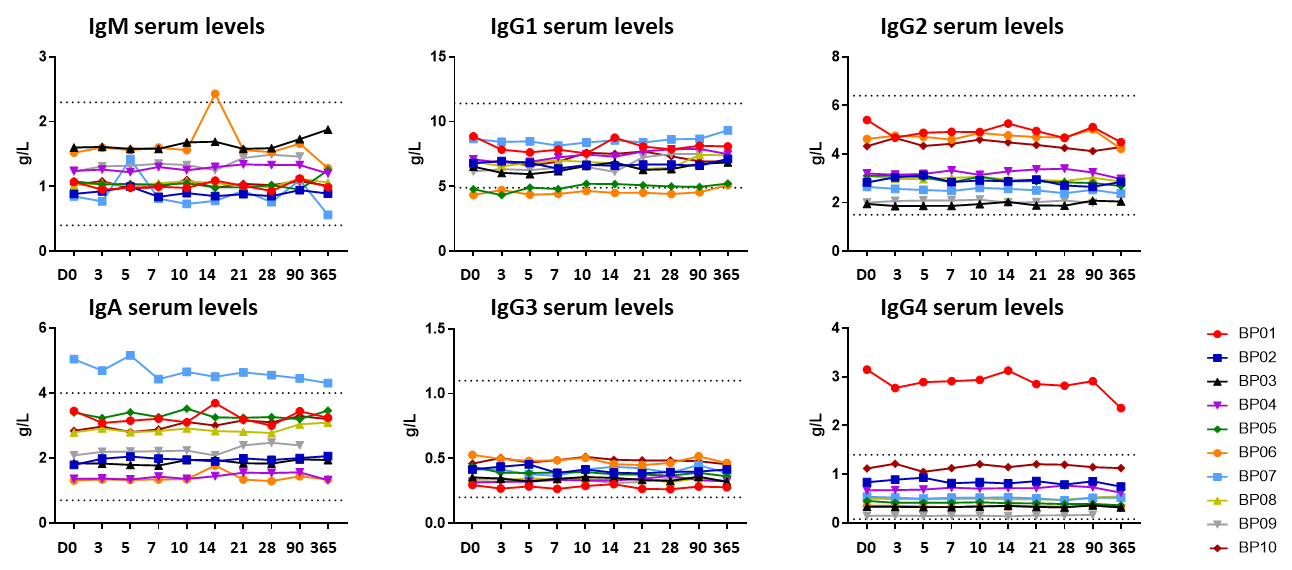


**Supplementary Figure 1.** **Serum Ig levels prior to and post-aP booster vaccination.**Total serum immunoglobulin levels (g/L) prior to and post-aP booster vaccination were determined by turbidimetry and levels of IgG subclasses (g/L) prior and post-Ap booster vaccination were determined by nephelometry. Dashed lines indicate the reference values for serum Ig levels used in the certified laboratory of Clinical Chemistry. D= Days after vaccination.

**
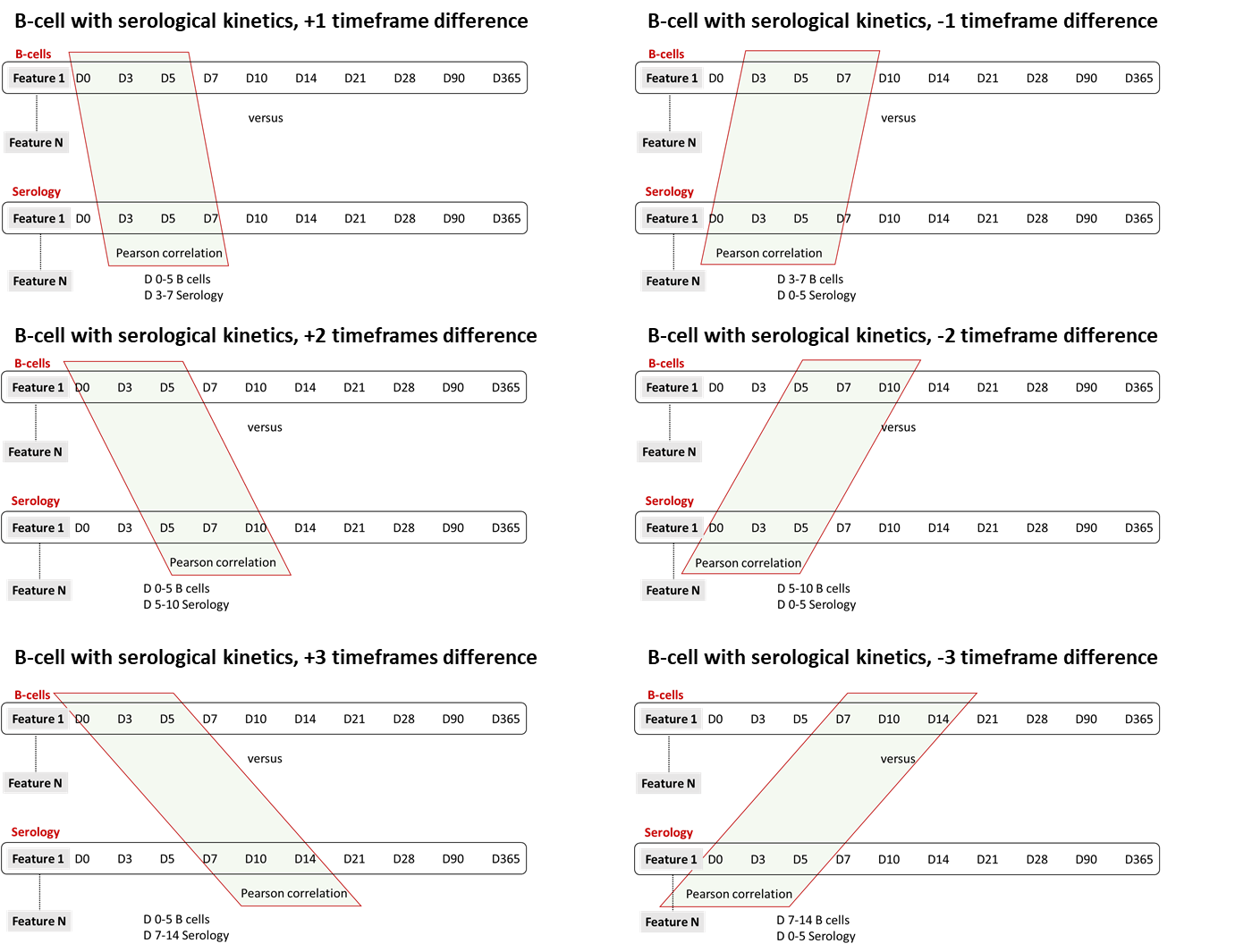
Supplementary Figure 2. Overview of the different comparisons over time performed between panels (comparison between earlier and later kinetics).** As example, the comparison between Ag-specific serum Ig levels (indicated as “serology”) and the B-cell compartment are shown. D= Days after vaccination.

**
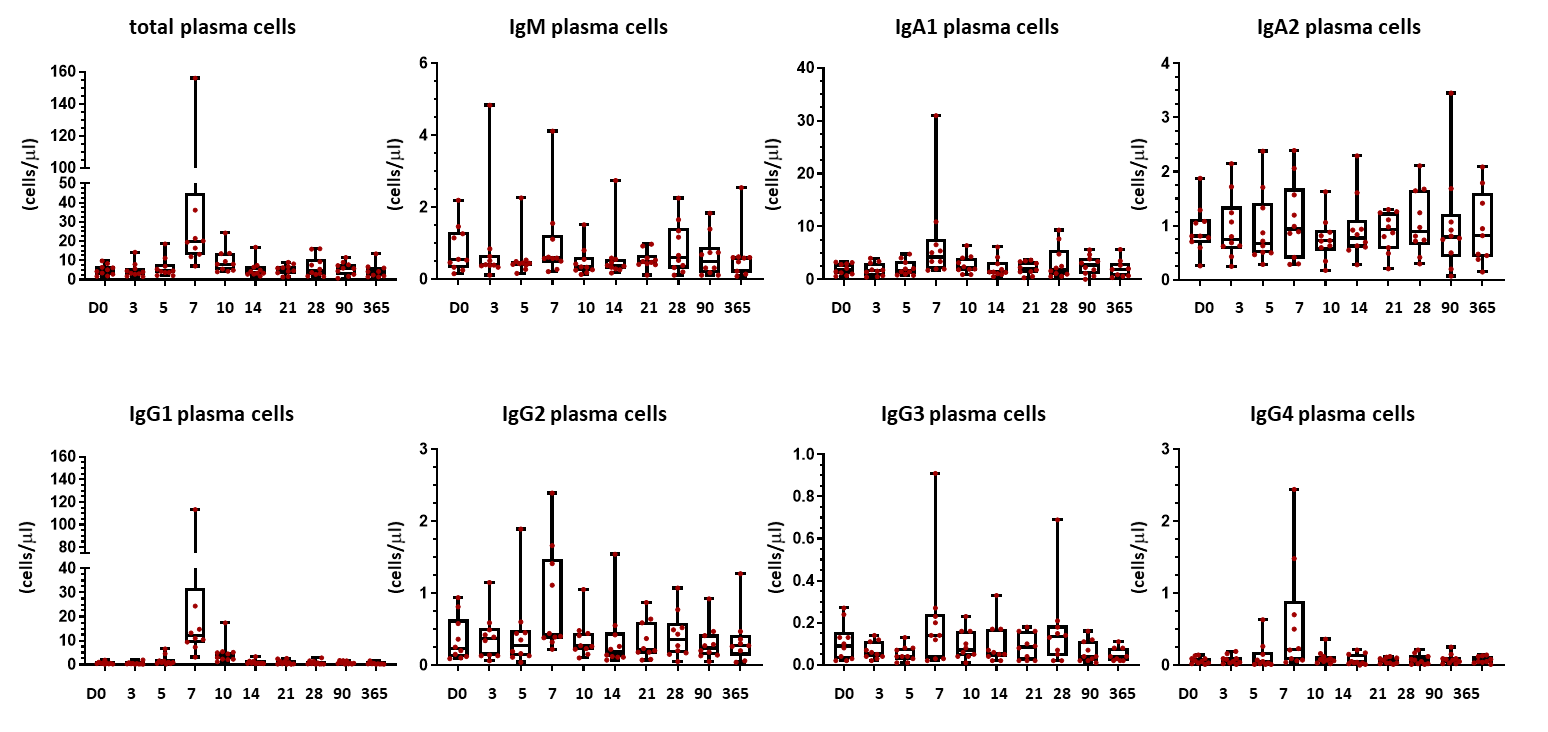
**

**Supplementary Figure 3. Kinetics in the plasma cell compartment post-Boostrix vaccination.** Boxplots indicating the fluctuations in cells/µl. Boxplots indicate min.-max., Q1, Q3 and median. Each dot represents one donor. D= Days after vaccination.

**
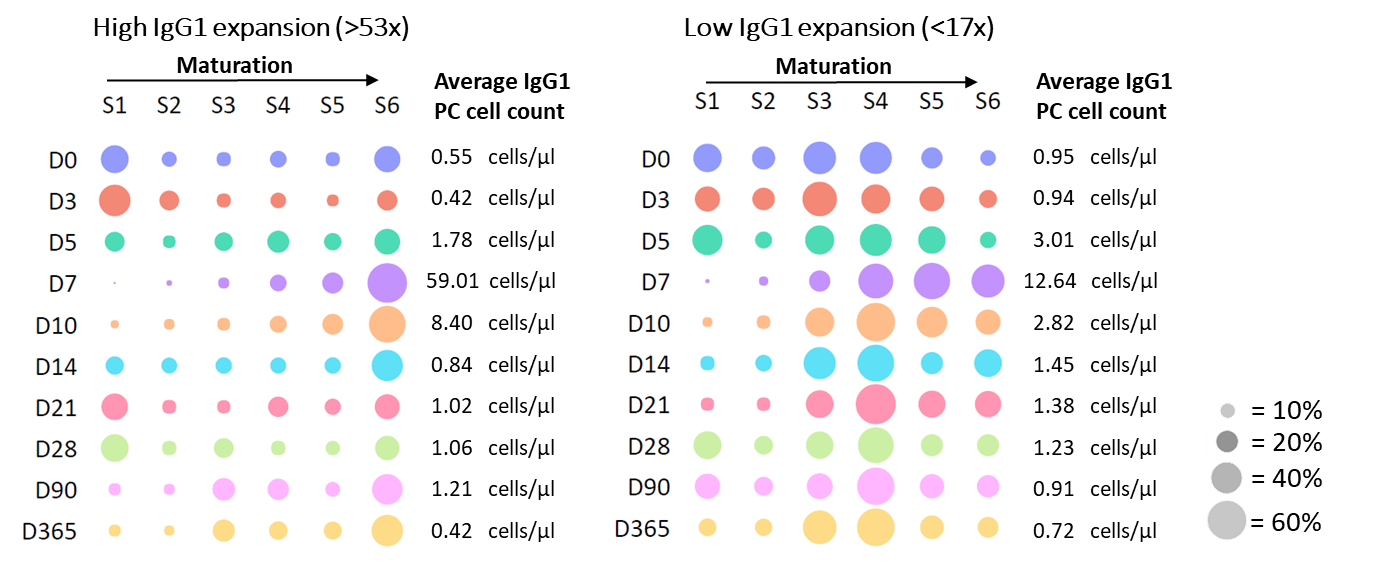
**

**Supplementary Figure 4. Maturation of IgG1 plasma cells in the 3 donors with the highest (ratio over baseline >53x) and lowest (ratio over baseline <17x) IgG1 plasma cell expansion**. Per time point the percentage of plasma cells in each maturation stage was plotted (total IgG1 plasma cells of all donors, grouped per time point). The size of the dot indicates the percentage of plasma cells in a given maturation stage (average of 3 donors). Cell count is shown at the right side of the plot (average of 3 donors). Bubble plots were generated using plotly python graphing library. D= Days after vaccination.

**
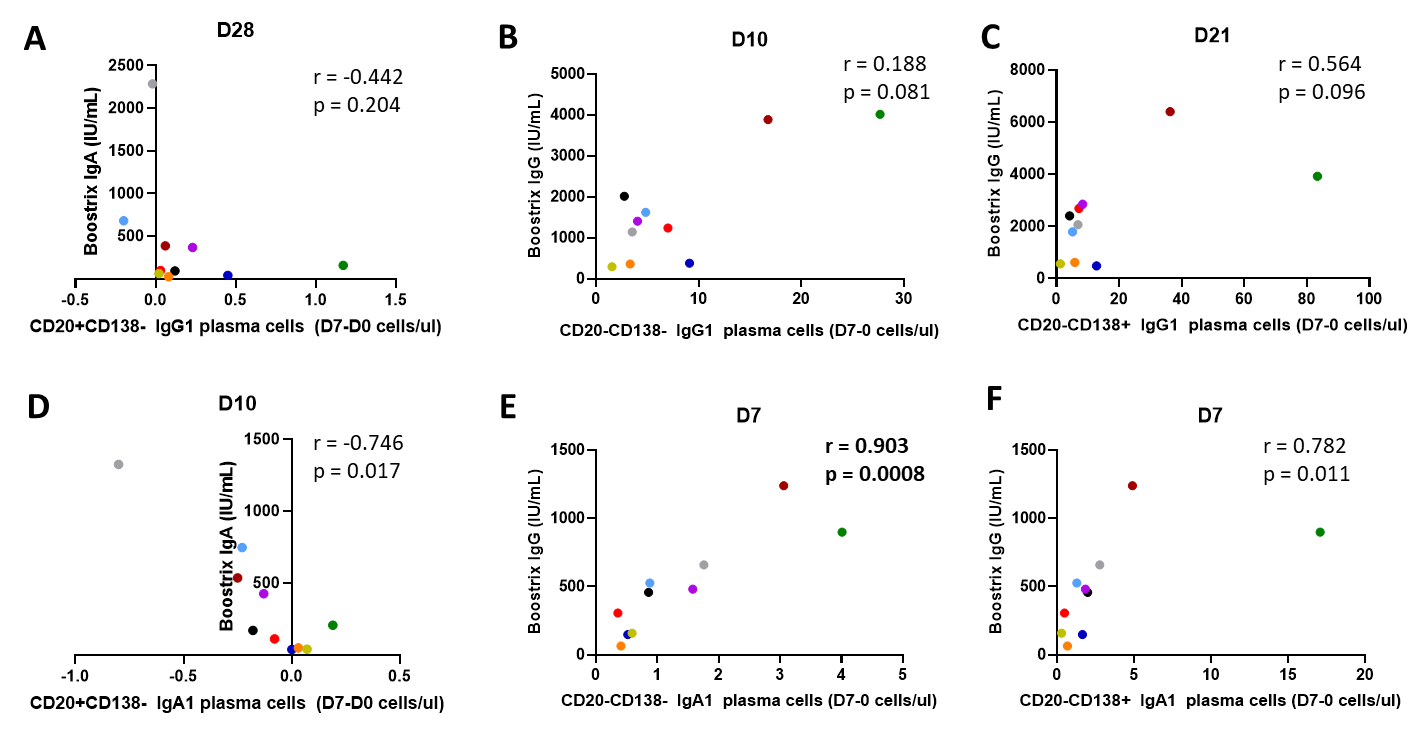
**

**Supplementary figure 5. Correlation between the absolute increase of different IgG1 and IgA1 plasma cell maturation stages at day 7 post-vaccination and the levels of Ag-specific serum IgG and IgA. For each maturation stage (defined based on CD20 and CD138 expression) only the strongest correlation is shown. A.** Strongest correlation found for CD20+CD138- IgG1 plasma cells. **B.** Strongest correlation found for CD20-CD138- IgG1 plasma cells. **C.** Strongest correlation found for CD20-CD138+ IgG1 plasma cells. **D.** Strongest correlation found for CD20+CD138- IgA1 plasma cells. **E.** Strongest correlation found for CD20-CD138- IgA1 plasma cells. **F.** Strongest correlation found for CD20-CD138+ IgA1 plasma cells. An FDR-corrected p-value of <0.0012 was considered significant. D = Days after vaccination.

**
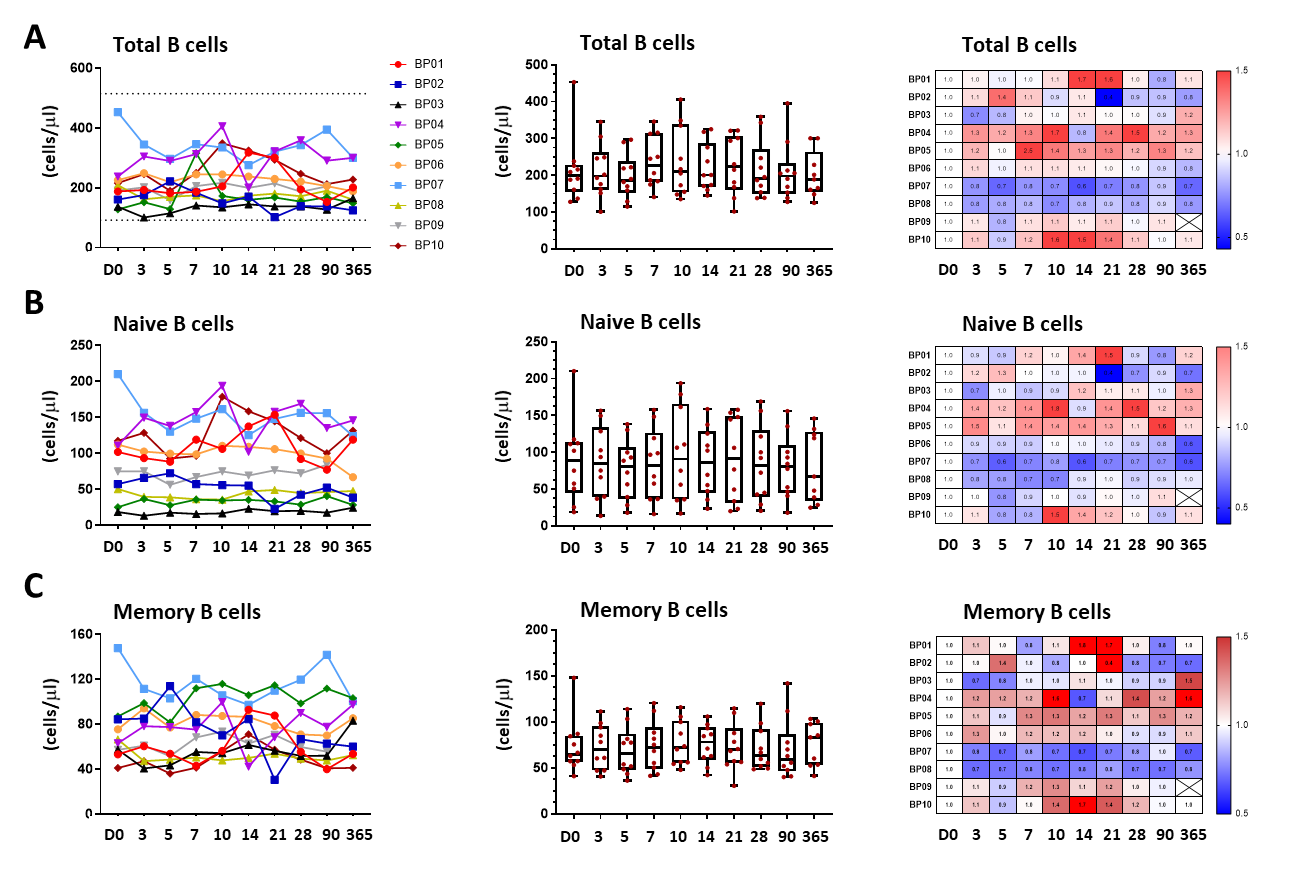
**

**Supplementary Figure 6. The expansion of total A. B cells, B. naive B cells and C. memory B cells upon vaccination with Boostrix in absolute cell numbers (graphs), grouped kinetics in absolute cell numbers (boxplots) and as ratio over baseline (heatmap).** Boxplots indicate min.-max., Q1, Q3 and median. Each dot represents one donor. D= Days after vaccination.

**
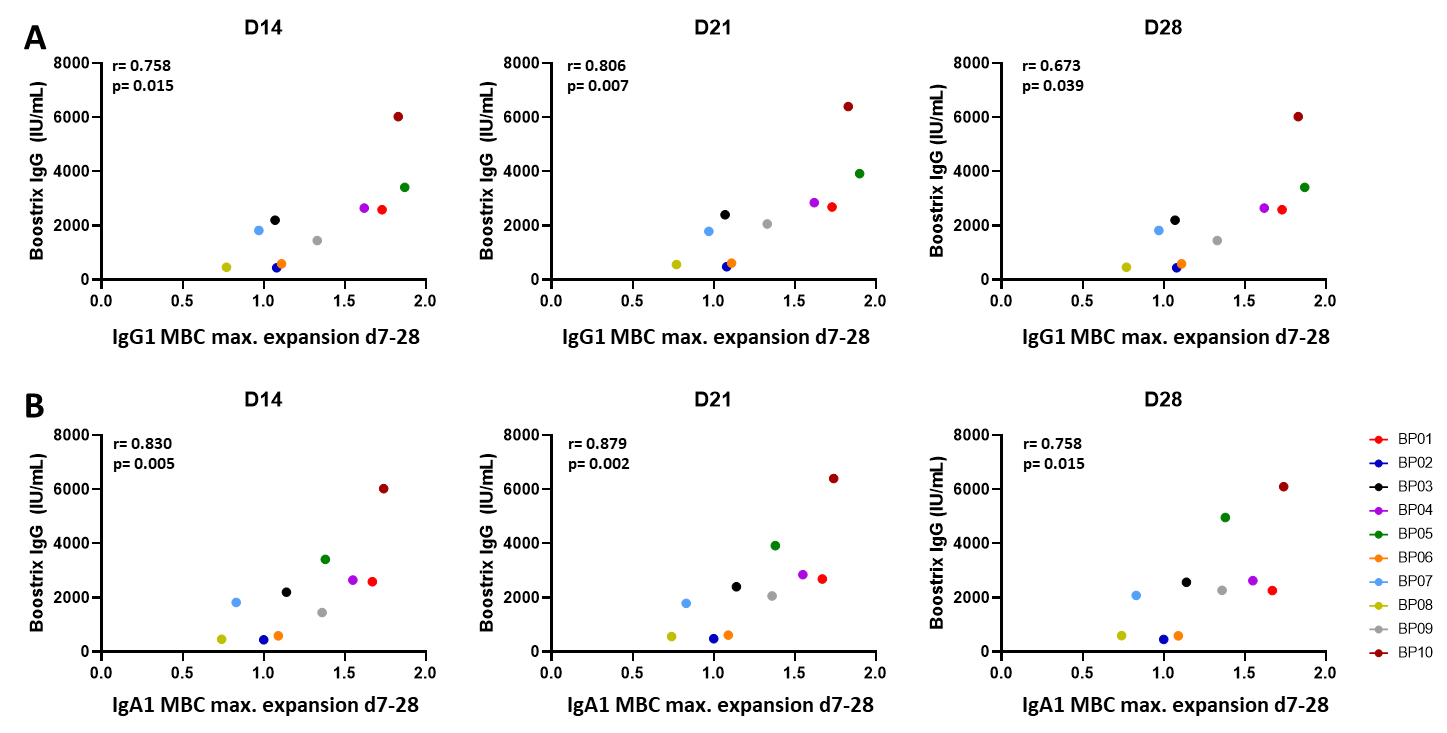
**

**Supplementary Figure 7. Correlation between maximum expansion of total B cells or leukocytes and vaccine-specific IgG as determined by Spearman’s rank correlation. A.** Correlation between the maximum expansion of total B cells (ratio over baseline day 7-28) and vaccine-specific IgG at d14, 21 and 28. **B.** Correlation between maximum expansion of total leukocytes (ratio over baseline day 7-28) and vaccine-specific IgG at d14, 21 and 28. An FDR-corrected p-value of <0.0075 was considered significant. D= Days after vaccination.

**
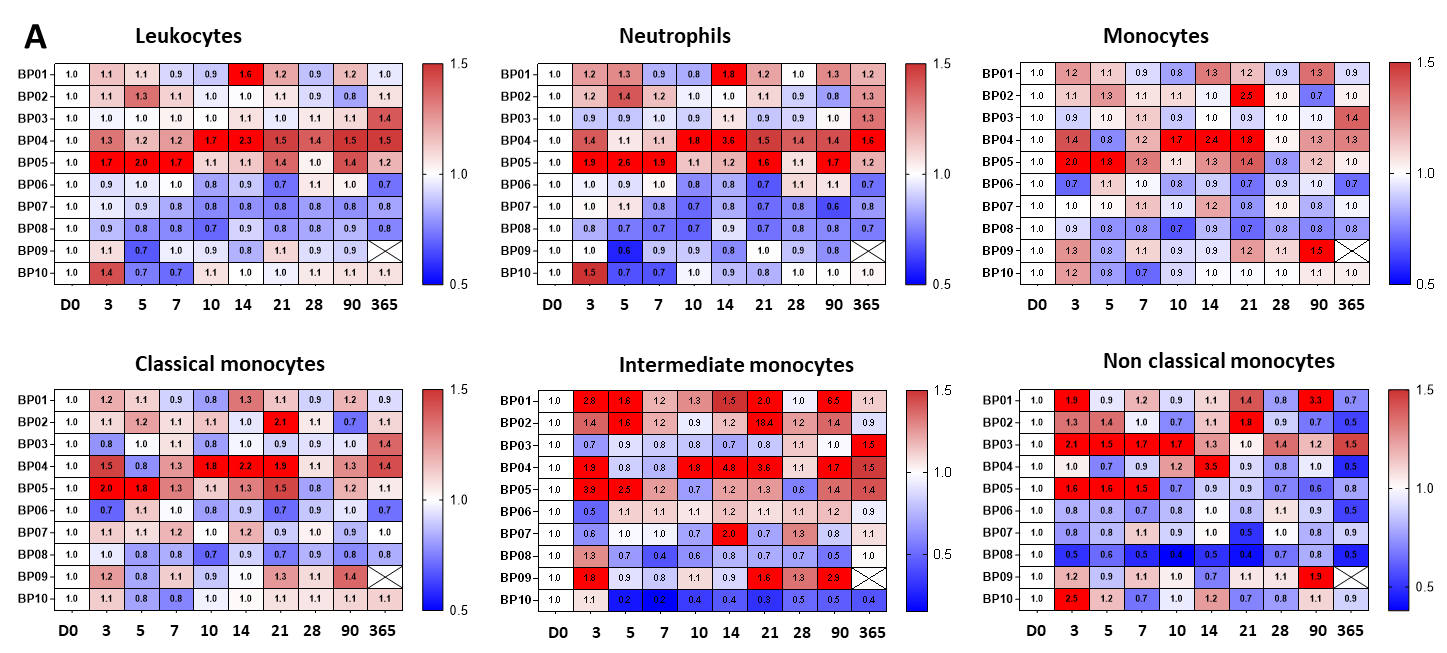
**

**
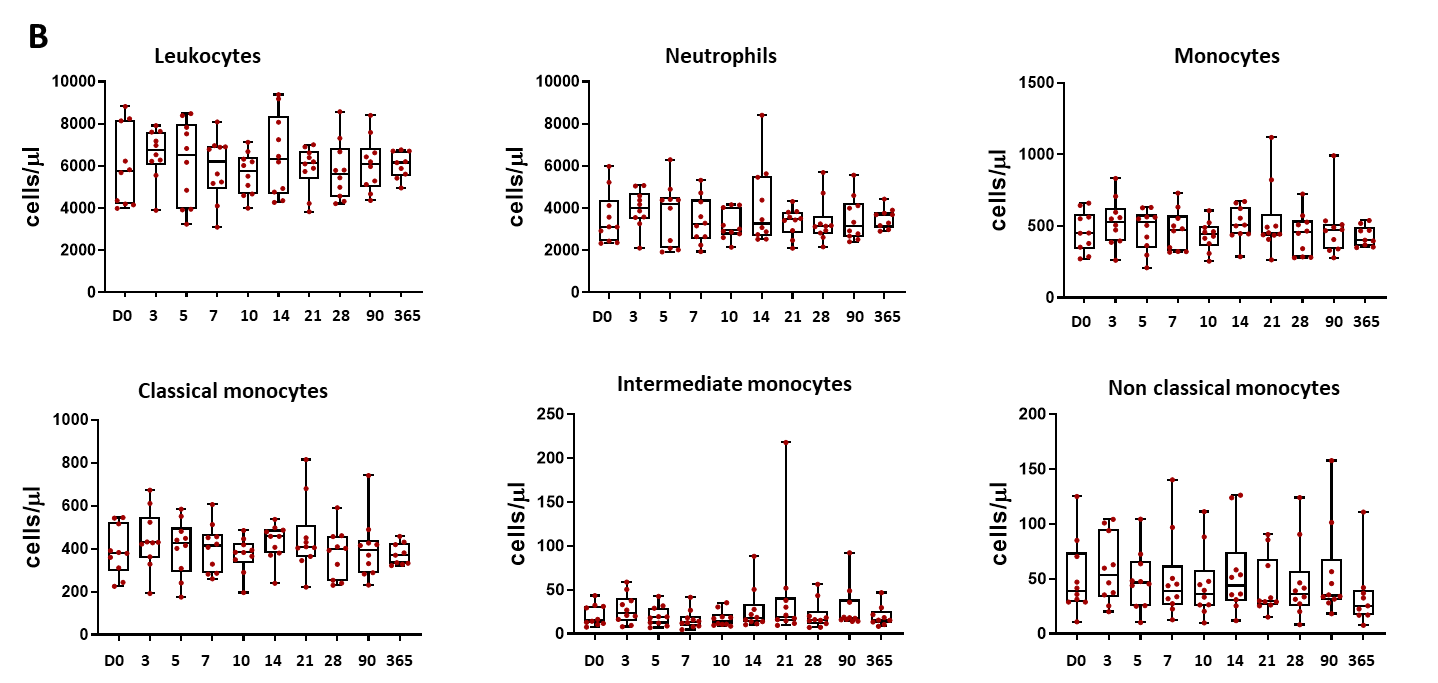
**

**Supplementary Figure 8. Most prominent fluctuations observed in innate immune cell subsets.** **A.** Heatmaps representing the expansion of leukocytes, neutrophils, monocytes and monocyte subsets in ratio over baseline. **B.** Boxplots indicating the fluctuations in cells/µl. Boxplots indicate min.-max., Q1, Q3 and median. Each dot represents one donor. D= Days after vaccination.
