# Supplementary material for: Highly Sensitive Flow Cytometry Allows Monitoring of Changes in Circulating Immune Cells in Blood After Tdap Booster Vaccination
Source: Front Immunol. 2021 Jun 10;12:666953. doi: 10.3389/fimmu.2021.666953 (PMC8223751; doi:10.3389/fimmu.2021.666953)
Supplement: Supplementary file 2 [file Table_1.pdf]

Supplementary Table 1: Exclusion criteria for this study.

Subjects who meet any of the following criteria will be excluded from participation in this study. The subject:	**Applicable to subject**
Has an auto-immune disease	**YES/NO**
Has an immune deficiency	**YES/NO**
Has a bleeding disorder	**YES/NO**
Underwent splenectomy	**YES/NO**
Receives immunosuppressive medication	**YES/NO**
Receives medication which influences blood clotting	**YES/NO**
Underwent active (clinically manifested) *Bordetella pertussis* infection at any point in life or had contact with such a person	**YES/NO**
Received vaccination against *Bordetella pertussis* in the past 10 years	**YES/NO**
Has Hb level below 8.4 g/dL (male) or below 7.8 g/dL (female)	**YES/NO**
Is pregnant or breast feeding	**YES/NO**
Had an allergic response to vaccination in the past	**YES/NO**
